# Cellular senescence induced by down-regulation of *PTBP1* correlates with exon skipping of mitochondrial-related gene *NDUFV3*

**DOI:** 10.1093/lifemedi/lnae021

**Published:** 2024-05-03

**Authors:** Yu Yang, Haimei Wen, Yuxin Li, Xin Zeng, Gang Wei, Zhenglong Gu, Ting Ni

**Affiliations:** Center for Mitochondrial Genetics and Health Research, Greater Bay Area Institute of Precision Medicine (Guangzhou), Fudan University, Guangzhou 511400, China; Collaborative Innovation Center of Genetics and Development, Human Phenome Institute, School of Life Sciences, Fudan University, Shanghai 200438, China; Collaborative Innovation Center of Genetics and Development, Human Phenome Institute, School of Life Sciences, Fudan University, Shanghai 200438, China; Collaborative Innovation Center of Genetics and Development, Human Phenome Institute, School of Life Sciences, Fudan University, Shanghai 200438, China; Collaborative Innovation Center of Genetics and Development, Human Phenome Institute, School of Life Sciences, Fudan University, Shanghai 200438, China; Collaborative Innovation Center of Genetics and Development, Human Phenome Institute, School of Life Sciences, Fudan University, Shanghai 200438, China; MOE Key Laboratory of Contemporary Anthropology, School of Life Sciences, Fudan University, Shanghai 200438, China; Center for Mitochondrial Genetics and Health Research, Greater Bay Area Institute of Precision Medicine (Guangzhou), Fudan University, Guangzhou 511400, China; Collaborative Innovation Center of Genetics and Development, Human Phenome Institute, School of Life Sciences, Fudan University, Shanghai 200438, China; Collaborative Innovation Center of Genetics and Development, Human Phenome Institute, School of Life Sciences, Fudan University, Shanghai 200438, China; National Clinical Research Center for Aging and Medicine, Huashan Hospital, Fudan University, Shanghai 200438, China; State Key Laboratory of Reproductive Regulation and Breeding of Grassland Livestock, Institutes of Biomedical Sciences, School of Life Sciences, Inner Mongolia University, Hohhot 010070, China

**Keywords:** cellular senescence, exon skipping, *PTBP1*, *NDUFV3*, mitochondria

## Abstract

As the most prevalent type of alternative splicing in animal cells, exon skipping plays an important role in expanding the diversity of transcriptome and proteome, thereby participating in the regulation of diverse physiological and pathological processes such as development, aging, and cancer. Cellular senescence serving as an anti-cancer mechanism could also contribute to individual aging. Although the dynamic changes of exon skipping during cellular senescence were revealed, its biological consequence and upstream regulator remain poorly understood. Here, by using human foreskin fibroblasts (HFF) replicative senescence as a model, we discovered that splicing factor PTBP1 was an important contributor for global exon skipping events during senescence. Down-regulated expression of *PTBP1* induced senescence-associated phenotypes and related mitochondrial functional changes. Mechanistically, PTBP1 binds to the third exon of mitochondrial complex I subunit coding gene *NDUFV3* and protects the exon from skipping. We further confirmed that exon skipping of *NDUFV3* correlates with and partially contributes to cellular senescence and related mitochondrial functional changes upon *PTBP1* knockdown. Together, we revealed for the first time that mitochondrial-related gene *NDUFV3* is a new downstream target for PTBP1-regulated exon skipping to mediate cellular senescence and mitochondrial functional changes.

## Introduction

Cellular senescence is a state in which the cells enter a permanent arrest of cell proliferation to prevent cancer and contribute to individual aging, and targeting cellular senescence can help combat aging [1–7]. Senescent cells exhibit several morphological and molecular markers, including enlarged and flattened cell morphology, increased senescence-associated β-galactosidase (SA-β-Gal) activity, down-regulation of proliferative markers like MKI67 and CDK1, increased expression of cell-cycle inhibitors (CDKN2A, CDKN1A, etc.), appearance of senescence-associated secretory phenotypes (SASPs) [4, 7–11]. Furthermore, senescent cells may also exhibit and partially induced by some other hallmarks of aging like mitochondrial dysfunction, which include decreased mitochondrial membrane potential, imbalanced energy metabolism homeostasis, increased reactive oxygen species (ROS), and mitochondrial superoxide (mtSOX) productions [4, 7, 12–14]. Cellular senescence can be divided into replicative and induced cellular senescence [9, 15]. Replicative senescence refers to the limited proliferative capacity of isolated body cells (such as primary fibroblasts) *in vitro*, which irreversibly stagnates after a certain number of passages, exhibiting significant cellular senescence phenotypes and some other hallmarks of aging, which was being called Hayflick limit [15–17]. Induced cellular senescence resulted from various stimulating factors (e.g. oncogene activation, genomic damage, UV radiation, oxidative stress, gene expression, and energy metabolism homeostasis imbalance) [15, 18, 19]. Exploring the characteristics and mechanisms of cellular senescence will aid the discovery of intervention targets for aging and age-related diseases [1, 2, 6].

Transcriptomic regulation is a core process of multi-biological processes, including senescence, and alternative splicing is an important component of transcriptomic regulation and a key contributor to expand the diversity of transcriptome and proteome. There are five major types of alternative splicing events, including exon skipping, alternative 5ʹ splice site, alternative 3ʹ splice site, mutually exclusive exon, and intron retention [20]. Among them, exon skipping is the most preventive type and has shown dynamic changes during cellular senescence. For example, a study has shown that regulatory RNA binding proteins may contribute to splicing alterations in replicative and induced cellular senescence, and exon skipping is the most prevent type of differential splicing events [21]. Furthermore, a study has also shown that during the aging processes of the female mouse hippocampus, exon skipping is the most prevalent alternative splicing event [22]. Although such studies have revealed that exon skipping is significantly changed in the cellular senescence process, the biological consequence and upstream regulator of exon-skipping events during cellular senescence remain poorly understood.

To explore the core factors that drive exon-skipping changes and their biological effects during cellular senescence, we analyzed poly(A)^+^ enriched RNA-seq data of human foreskin fibroblasts (HFF) replicative senescence model and found hundreds of differential exon-skipping events during HFF senescence. We further found that eight RNA binding proteins (RBPs), including PTBP1, bind to these differentially spliced exons. Knockdown of *PTBP1* promotes a wide range of exon-skipping changes, significant senescence-associated phenotypes, and related mitochondrial functional changes. In particular, we found that *PTBP1* downregulation promotes exon skipping of *NDUFV3*, which encodes a subunit of mitochondrial complex I, leads to cellular senescence and related mitochondrial functional changes. Multiple lines of evidence support that PTBP1-mediated exon skipping of *NDUFV3* exists in various cellular senescence models and cancer types. Altogether, we discovered that down-regulation of splicing factor PTBP1 induced cellular senescence and related mitochondrial functional changes, which is correlated with the promoting exon skipping of *NDUFV3*.

## Results

### Exon skipping is prevalent during HFF cellular senescence

**Figure 1.**
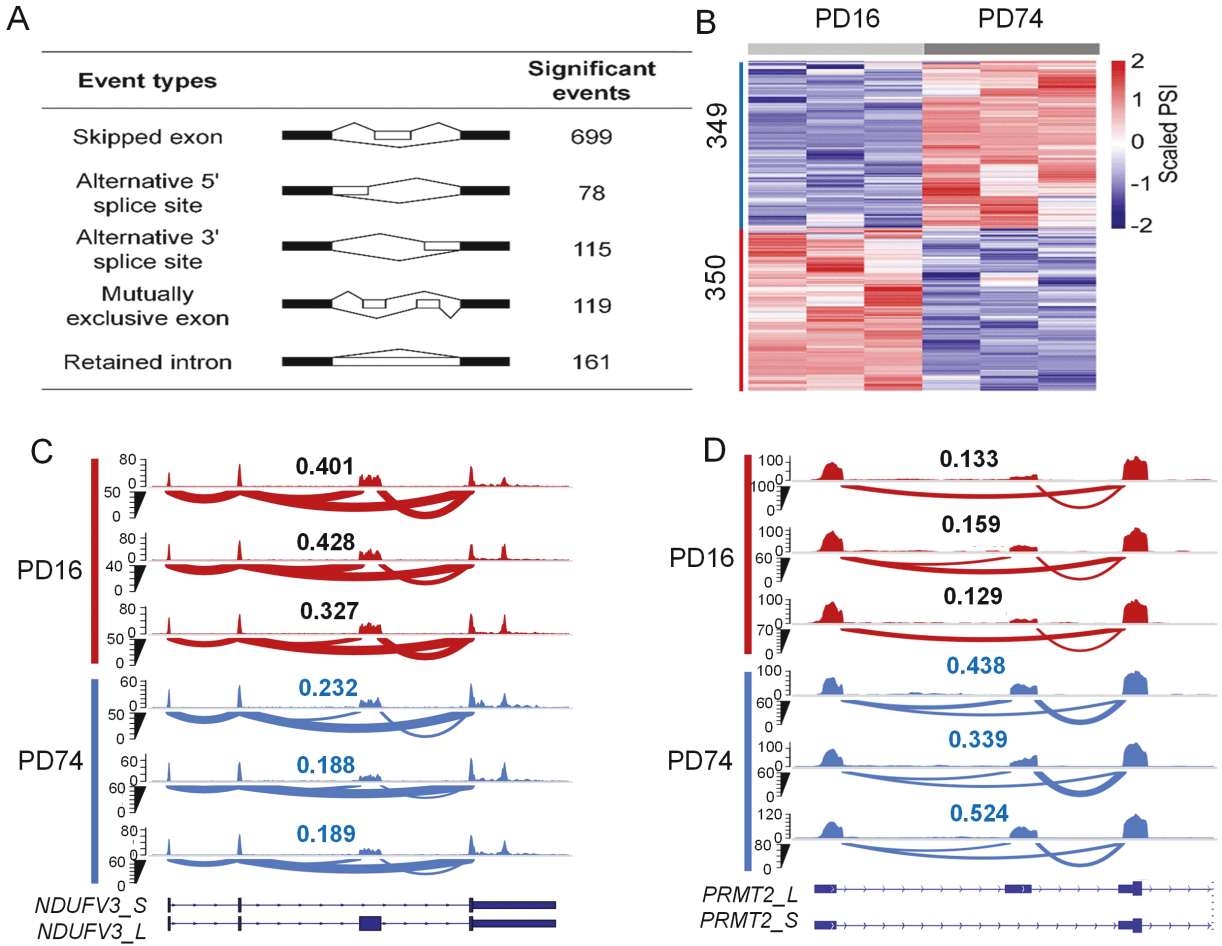
Exon skipping is prevalently changed during HFF cellular senescence. (A) Classification statistics of differential alternative splicing events before and after HFF cellular senescence. (B) Heatmap of differential exon-skipping events before and after HFF cellular senescence. The numbers of differentially skipped exons were shown on the left. PSI, percent spliced in. Each time point has three biological replicates. (C) Wiggle plots showing up-regulated exon-skipping events of *NDUFV3* supported by poly(A)^+^ RNA-seq. The PSI index is shown on the top of each sample. Three biological replicates were shown for each time point. (D) Wiggle plots showing downregulated exon-skipping events of *PRMT2* supported by poly(A)^+^ RNA-seq. The PSI index is shown on the top of each sample. Three biological replicates were shown for each time point.

To investigate whether global exon skipping changes exist in cellular senescence, we used published poly(A)^+^ RNA-sequencing (RNA-seq) data derived from human foreskin fibroblast (HFF) consisting of five different time points, each of which has three biological replicates [23]. We selected a senescent time point (population doubling number 74, or PD74) and compared it to the young cells (PD16). The widely used rMATS method was applied for differential alternative splicing analysis [24]. We found that there were significant differences in alternative splicing changes during replicative senescence of HFF, and the most dominant type (699/1172) is exon skipping (Fig. 1A). About 49.9% exon-skipping events (349) were upregulated, and 50.1% (350) were downregulated in senescent HFF cells (Fig. 1B). The skipping of 3rd exon in *NDUFV3* served as an example for upregulated exon-skipping events (Fig. 1C), while the skipping of 2nd exon in *PRMT2* served as an example for down-regulated exon-skipping events (Fig. 1D). NDUFV3 is a subunit of the important NADH-ubiquinone oxidoreductase complex (mitochondrial complex I), and the 3rd exon of *NDUFV3* is relatively conserved among vertebrates. Additionally, the exon-preserved transcript of *NDUFV3* (*NDUFV3_L*) is more tend to expressed in proliferative cells, while the exon-skipped transcript of *NDUFV3* (*NDUFV3_S*) is more expressed in proliferation-arrested heart cells [25–28]. Therefore, the increase of exon skipping of *NDUFV3* may contribute to cellular senescence and related mitochondrial dysfunction.

### Regulated skipped exons during HFF senescence have distinct RBP-binding features

**Figure 2.**
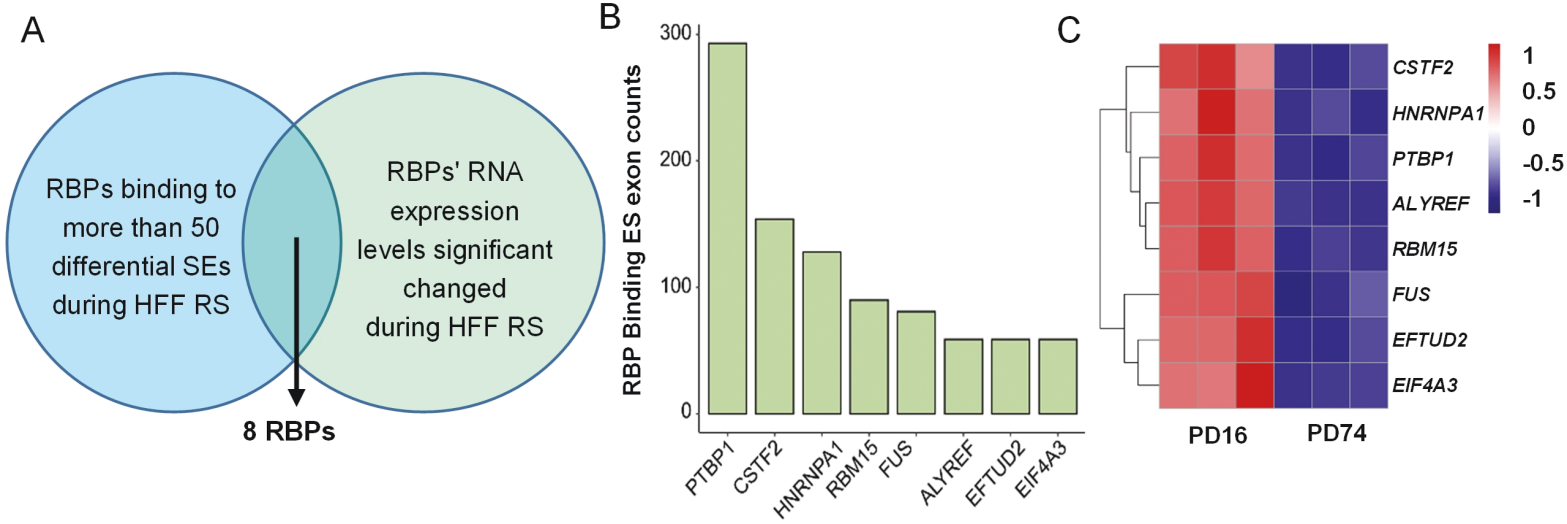
Regulated skipped exons during senescence have distinct RBP-binding features. (A) Analysis processes of RBPs that potentially regulate the senescence-associated exon skipping of HFF replicative cells. (B) Binding counts of eight RBPs in panel A at those cellular senescence-related differential exons. (C) Heatmap of expression levels of genes encoding eight RBPs during HFF replicative senescence.

We next explored mediators of the above exon-skipping events during HFF senescence. RBPs play important roles in alternative splicing including exon skipping, and some studies have reported that most RBP-coding genes are down-regulated in various cell senescence models [21]. Therefore, we speculated that down-regulation of certain RBPs may play important roles in exon skipping changes during HFF replicative senescence. In order to find the core RBPs that mediate exon skipping during HFF senescence, on the one hand, we combined the binding site information of 220 RBPs integrated by POSTAR 3.0 [29, 30] to explore the binding features of RBPs on those changed exons. On the other hand, by integrating the gene expression profile data of HFF senescence, we can find which RBP-coding genes are significantly down-regulated during HFF replicative senescence (Fig. 2A). This integration analysis leads to the discovery of eight RBPs (PTBP1, CSTF2, HNRNPA1, RBM15, FUS, ALYREF, EFTUD2, EIF4A3), which not only have binding signals in those differentially spliced exon regions (Fig. 2B) but also show significantly down-regulated expression in senescent cells compared to the younger ones (Fig. 2C). Among them, PTBP1 ranks the top regarding the binding counts to these differentially spliced exons (Fig. 2B). Given PTBP1 plays important roles in regulating intron retention and exon inclusion that containing transposable elements [31, 32] and also functions in cell proliferation and neuron differentiation [32, 33], we speculated that downregulation of PTBP1 may play a more important role in promoting cellular senescence via exon skipping than others candidate RBPs.

### Downregulation of RNA binding protein PTBP1 mediates widespread exon skipping changes including *NDUFV3*’*s* 3rd exon

**Figure 3.**
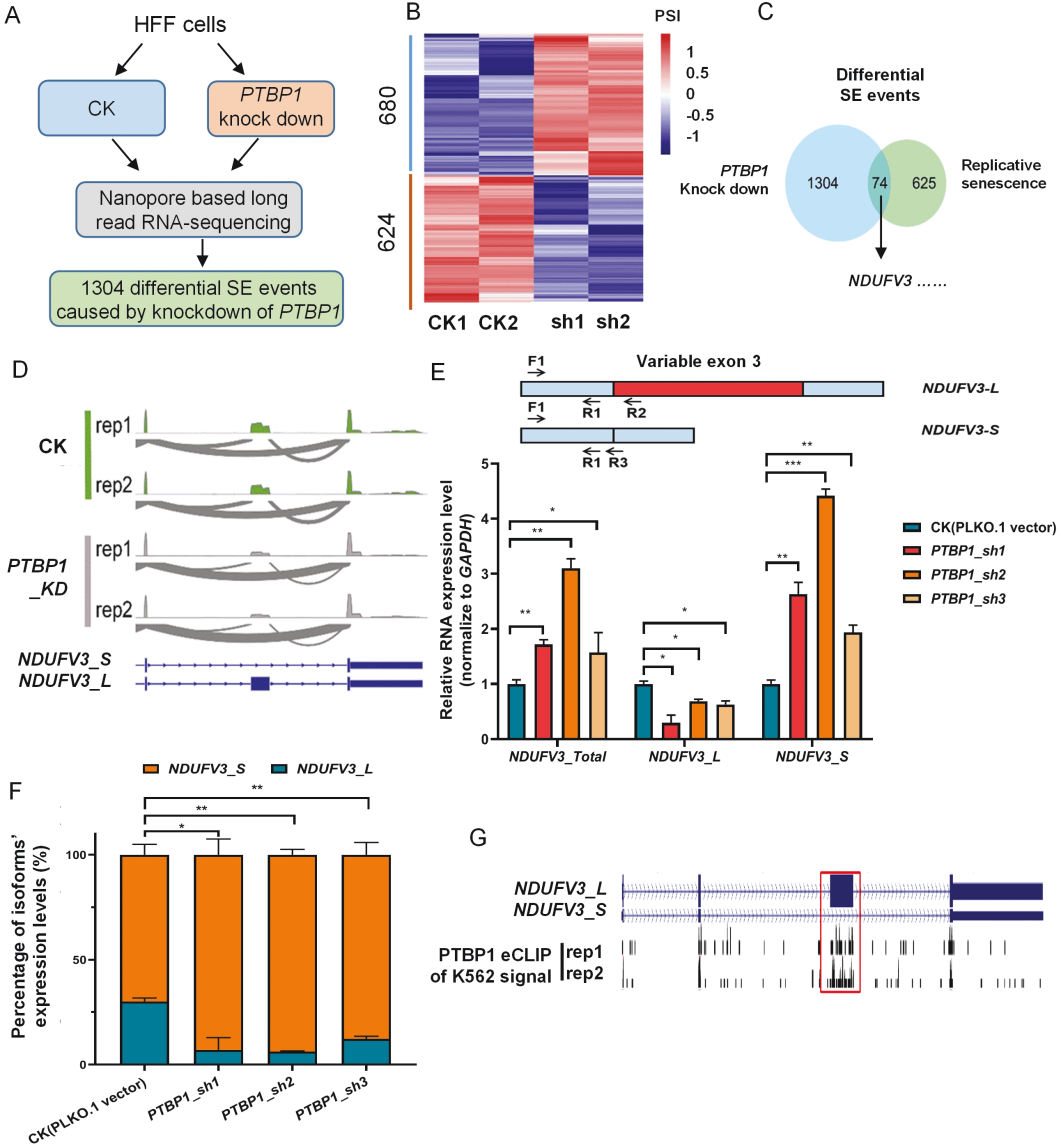
*PTBP1* down-regulation mediates widespread exon skipping changes including *NDUFV3*’s 3rd exon. (A) Workflow to identify differential exon-skipping events before and after knockdown of *PTBP1* in HFF cells. (B) Heatmap of differential exon-skipping events before and after *PTBP1* knock down in HFF cells. (C) Venn diagram showing overlap of differential exon-skipping events among replicative senescence and *PTBP1* knock down in HFF cells. (D) Wiggle plots showing upregulated exon-skipping event of *NDUFV3*’s 3rd exon supported by poly(A)^+^ RNA-seq. (E) Primer pairs design mode diagram and verification of gene’s and transcripts’ expression levels of *NDUFV3* before and after *PTBP1* knock down in HFF cells by qRT-PCR (Note: Primer pair of F1/R1 was used to detect the total RNA expression level of *NDUFV3*, F1/R2 was used to detect the RNA expression level of *NDUFV3_L*, and F1/R3 was used to detect the RNA expression level of *NDUFV3_S*. The relative expression level of *GAPDH* was used as an internal reference, and the relative expression levels of *NDUFV3_Total*, *NDUFV3_L*, and *NDUFV3_S* were all normalized to their expression levels in CK samples). (F) Percentage of the expression of the two isoforms of *NDUFV3* before and after *PTBP1* knock down in HFF cells by qRT-PCR results of Fig. 3E. (G) Wiggle plots show binding signals of PTBP1 among *NDUFV3* transcribed pre-mRNA by eCLIP-seq. *, *P* < 0.05; **, *P* < 0.01; ***, *P* < 0.001, *t*-test.

In order to systematically explore the effect of PTBP1’s down-regulation on alternative splicing (especially exon skipping), we performed Oxford Nanopore (ONT) based long read RNA sequencing before and after knockdown of *PTBP1* in HFF cells, then we applied DRIMSeq [34] to analyze the differences of alternative splicing events. We found that the knockdown of *PTBP1* by two short hairpin RNAs (shRNAs) in HFF cells all led to a wide range of exon skipping changes (Fig. 3A), and about 52.15% common exon skipping events (680/1304) were upregulated, and 47.85% common exon-skipping events (624/1304) were downregulated in *PTBP1* downregulated HFF (Fig. 3B). By overlapping with the differential exon skipping events, and combining the binding signals of PTBP1 on related differential exons by POSTAR 3.0, we found that downregulation of *PTBP1* may directly explain about 10.6% (74/699) of the differential exon-skipping events in the process of HFF replicative cellular senescence, and the exon-skipping event of *NDUFV3*’s 3rd exon is one of them (Fig. 3C, Table S1). Since NDUFV3 is a subunit of the important NADH-ubiquinone oxidoreductase complex (mitochondrial complex I), and the differential expression between *NDUFV3_L* and *NDUFV3_S* has significant tissue specificity [25–27], and the exon-skipping level of *NDUFV3* was considerably changed upon *PTBP1* knockdown, we hypothesized that the exon skipping of *NDUFV3* regulated by PTBP1 may play a critical role in cellular senescence and related mitochondrial functional changes. Therefore, we conducted an in-depth investigation on the exon skipping of *NDUFV3*.

As shown in Fig. 3D and Fig. S1, the RNA-seq sashimi plots showed that knockdown of *PTBP1* resulted in a significant increase of exon-skipping event of *NDUFV3*’s 3rd exon in HFF and HEK293T cells (*NDUFV3_S*’s expression increased and *NDUFV3_L*’s expression decreased, that is, the proportion of *NDUFV3_S/NDUFV3* increased significantly). Reverse transcription followed by quantitative real-time polymerase chain reaction (qRT-PCR) also confirmed such changes upon PTBP1 knockdown in HFF cells (Fig. 3E and 3F). In addition, public enhanced crosslinking immunoprecipitation (eCLIP) data revealed that PTBP1 could bind to the RNA region corresponding to the 3rd exon of *NDUFV3* (Fig. 3G), and the related region also has the binding motif sequences (CUCU rich) of PTBP1 (Fig. S2) [35]. These above results support that PTBP1 protects the 3rd exon of *NDUFV3* from skipping via direct binding.

### Down-regulation of *PTBP1* contributes to cellular senescence and related mitochondrial functional changes

As mentioned above, *PTBP1* is significantly down-regulated during the replicative senescence processes of HFF and other cells, and its downregulation leads to widespread exon skipping changes including mitochondrial complex I subunit coding gene *NDUFV3*; we therefore examined whether knocking down *PTBP1* could lead to cellular senescence and related mitochondrial functional changes.

**Figure 4.**
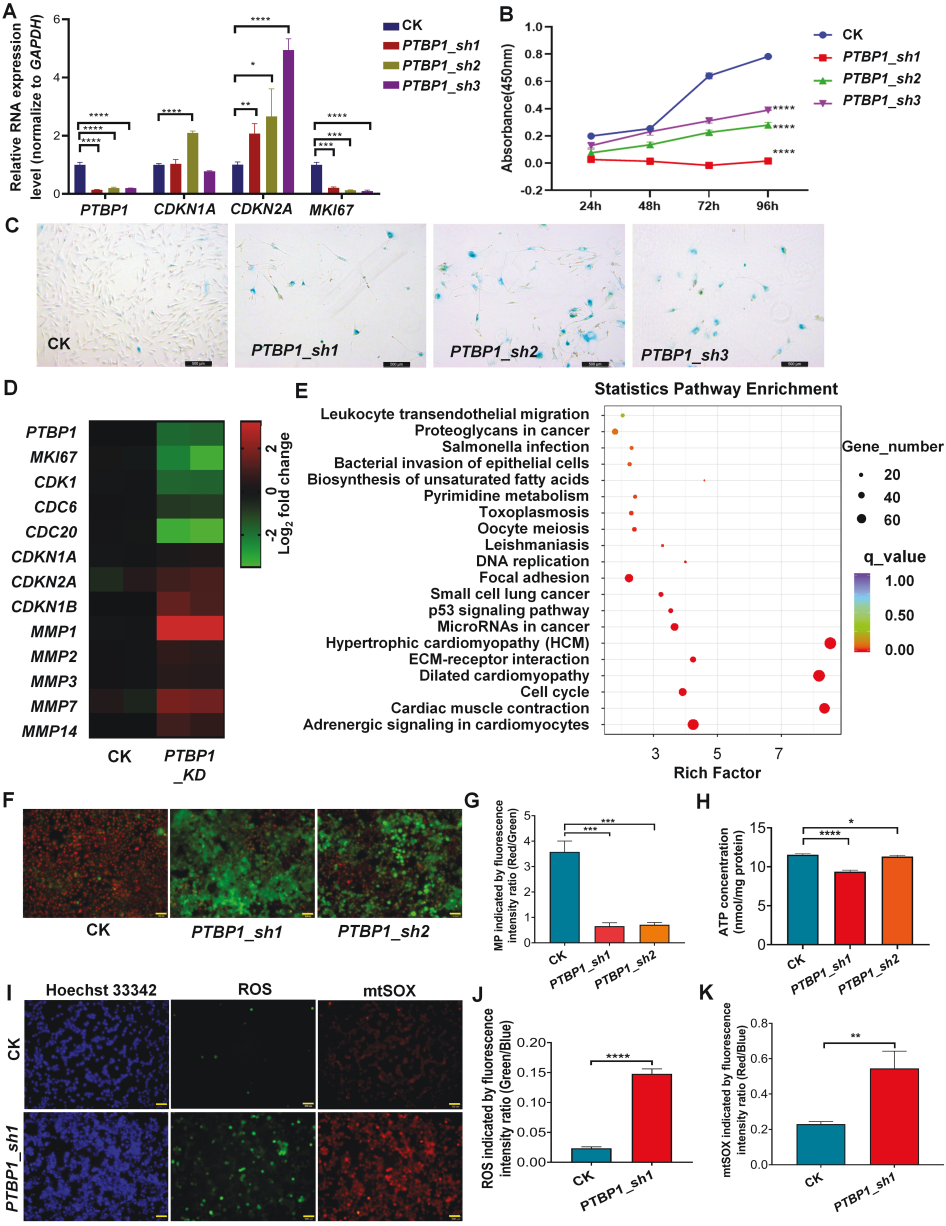
Knockdown of *PTBP1* leads to cellular senescence and mitochondrial-related functional changes. (A) Gene expression evaluation of *PTBP1, CDKN1A, CDKN2A*, and *MKI67* before and after *PTBP1* knockdown in HFF cells by qRT-PCR. (B) The proliferation rate of control (CK) and *PTBP1*_KD HFF cells was measured by CCK-8 assay. (C) SA-β-Gal staining of CK and *PTBP1*_KD HFF cells. Bars, 500 μm. (D) Heatmap showing *PTBP1* and some other cellular senescence-related genes’ relative expression levels in CK and *PTBP1*_KD HFF cells by ONT-based long-read sequencing. (E) KEGG pathway enrichment analysis of differentially expressed genes before and after knockdown of *PTBP1* by ONT-based long-read sequencing. (F) Fluorescence staining images by JC1 indicate the mitochondrial membrane potential were loss after *PTBP1* knock down in HEK293T cells (Note: Red represents high mitochondrial membrane potential, and green represents low mitochondrial membrane potential). Bars, 500 μm. (G) Quantitative measure of mitochondrial membrane potential based on fluorescence intensity ratio (Red/Green) by JC1 staining in CK and *PTBP1*_KD HEK293T cells. (H) ATP concentration in CK and *PTBP1*_KD HEK293T cells. (I) Fluorescence staining images of chromatin, intracellular ROS, and mtSOX in CK and *PTBP1*_KD HEK293T cells. Bars, 500 μm. (J) The quantitative measure of intracellular reactive oxygen species (ROS) relative amount based on fluorescence intensity ratio (Green/Blue) in CK and *PTBP1*_KD HEK293T cells. (K) Quantitative measure of intracellular mitochondrial superoxide (mtSOX) relative amount based on fluorescence intensity ratio (Red/Blue) in CK and *PTBP1*_KD HEK293T cells. *, *P* < 0.05; **, *P* < 0.01; ***, *P* < 0.001; ****, *P* < 0.0001, *t*-test. For colour version of this figure refer to online source.

To address this question, we applied three shRNAs targeting *PTBP1* to perform the RNA inference in HFF and HEK293T cells. We found that knocking down *PTBP1* led to significant cellular senescence phenotypes (Fig. 4 and Fig. S3), including up-regulation of *CDKN1A* and *CDKN2A* (or *CDKN2B*) expression levels (Fig. 4A and Fig. S3A), down-regulation of *MKI67* expression levels (Fig. 4A), reduced cell proliferation rate (Fig. 4B and Fig. S3B), and a significant increase in the proportion of SA-β-Gal (senescence-associated β-galactosidase)-positive cells and enlarge of cell morphology (Fig. 4C and Fig. S3C). To explore the global molecular phenotype changes upon *PTBP1* knockdown, we carried out ONT-based long read RNA sequencing in HFF cells as above. Heatmaps of differentially expressed genes upon *PTBP1* knockdown further confirmed reduced expression of such proliferative marker genes like *MKI67*, *CDK1*, *CDC6*, and *CDC20*, together with increased expression of cell-cycle inhibitors like *CDKN1A*, *CDKN2A*, *CDKN1B*, and gene family members of matrix metalloproteinases (MMPs) that belong to SASP (Fig. 4D). KEGG pathway enrichment analysis showed that these differentially expressed genes were enriched in senescence-related pathways such as cell cycle, DNA replication, and p53 signaling pathway (Fig. 4E) [6]. Furthermore, we found that the knockdown of *PTBP1* can significantly reduce mitochondrial membrane potential (Fig. 4F and 4G) and slightly decrease intracellular ATP concentration (Fig. 4H), while obviously increasing intracellular reactive oxygen species (ROS) concentration and mitochondrial superoxide (mtSOX) concentration (Fig. 4I–K). Therefore, down-regulation of *PTBP1* contributes to cellular senescence and related mitochondrial functional changes.

### Exon skipping of *NDUFV3* contributes to cellular senescence and related mitochondrial functional changes

**Figure 5.**
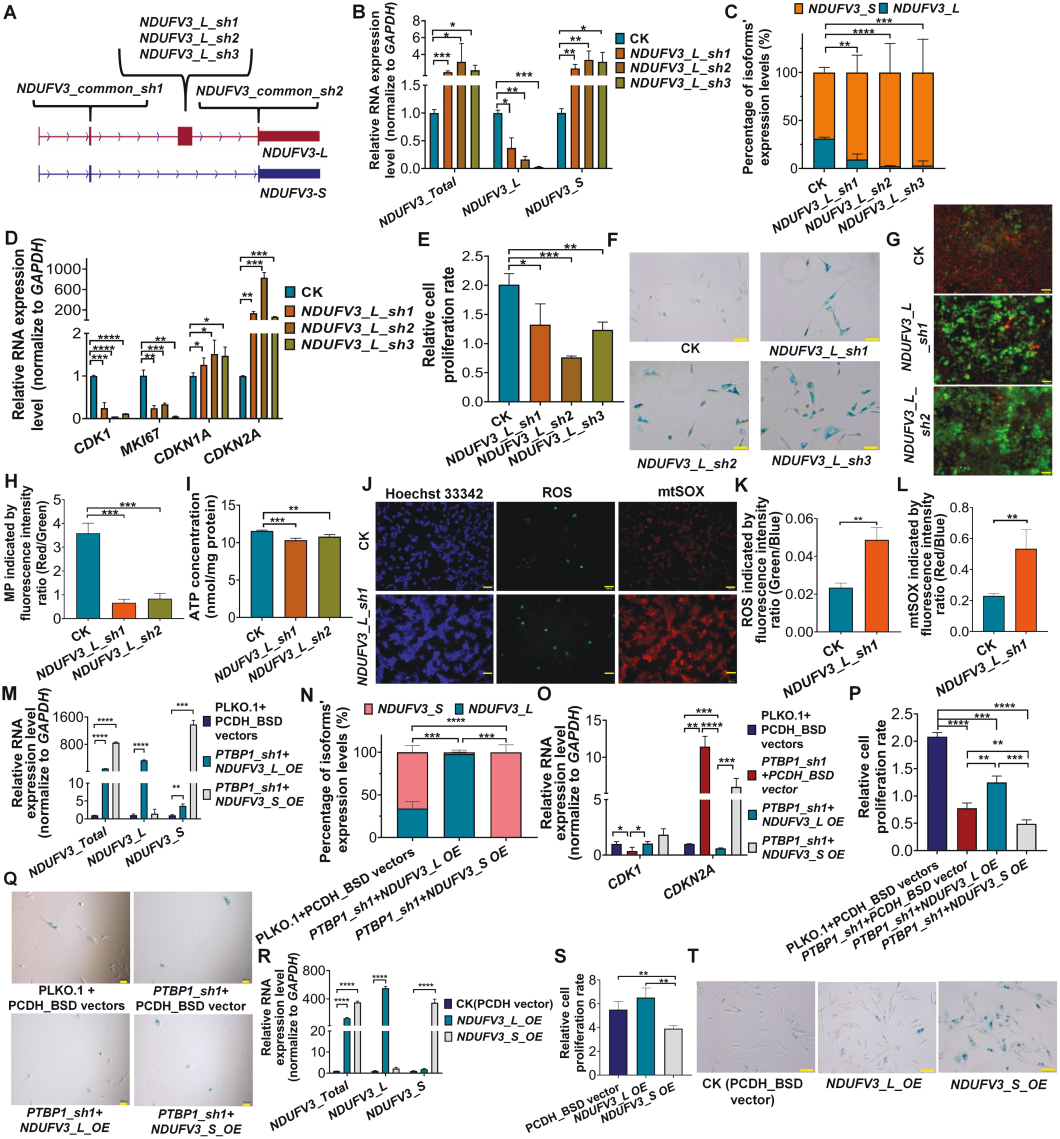
Exon skipping of *NDUFV3* contributes to cellular senescence and mitochondrial-related functional changes. (A) Diagram of shRNAs targeting *NDUFV3*’s common and 3rd exons. (B) Verification of *NDUFV3* gene and transcript expression levels in HFF cells before and after *NDUFV3_L* knockdown by qRT-PCR. Note: Same primers as Fig. 3E. (C) Percentage of *NDUFV3* isoforms’ expression in HFF before and after *NDUFV3_L* knockdown by results of Fig. 5B. (D) Gene expression evaluation of *CDK1*, *MKI67*, *CDKN1A*, and *CDKN2A* before and after *NDUFV3_L* knock down in HFF by qRT-PCR. (E) Relative proliferation rate of control (CK) and *NDUFV3_L*_KD HFF measured by CCK-8 assay. (F) SA-β-Gal staining of CK and *NDUFV3_L*_KD HFF cells. Bars, 200 μm. (G) Fluorescence staining images indicate the mitochondrial membrane potential of CK and *NDUFV3_L*_KD HEK293T cells by JC1. Bars, 500 μm. (H) Quantitative measure of MP based on fluorescence ratio (Red/Green) of Fig. 5G in CK and *NDUFV3_L_*KD HEK293T. (I) ATP concentration in CK and *NDUFV3_L*_KD HEK293T. (J) Fluorescence staining images of chromatin (Hoechst 33342), intracellular ROS and mtSOX in CK and *NDUFV3_L*_KD HEK293T. Bars, 500 μm. (K) Quantitative measure of intracellular ROS based on fluorescence ratio (Green/Blue) of Fig. 5J in CK and *NDUFV3_L*_KD HEK293T. (L) The quantitative measure of intracellular mtSOX based on fluorescence ratio (Red/Blue) of Fig. 5J in CK and *NDUFV3_L*_KD HEK293T. (M) Evaluation of gene and transcripts expression of *NDUFV3* before and after *NDUFV3_L* and *NDUFV3_S* overexpressed in *PTBP1*_KD HFF by qRT-PCR. (N) Percentage of *NDUFV3*’s two isoforms’ expression before and after *NDUFV3_L* and *NDUFV3_S* overexpressed in *PTBP1*_KD in HFF by results of Fig. 5M. (O) Expression evaluation of *CDK1, CDKN2A* before and after *NDUFV3_L* and *NDUFV3_S* overexpressed in *PTBP1*_KD HFF by qRT-PCR. (P) Relative proliferation rate measured by CCK-8 assay before and after *NDUFV3_L* and *NDUFV3_S* overexpressed in *PTBP1*_KD HFF cells. (Q) SA-β-Gal staining of HFF before and after *NDUFV3_L* and *NDUFV3_S* overexpressed on the basis of *PTBP1* knockdown. Bars, 100 μm. (R) Gene and transcripts expression levels evaluation of *NDUFV3* before and after *NDUFV3_L* and *NDUFV3_S* overexpressed in HFF by qRT-PCR. (S) Relative proliferation rate of control (PCDH_BSD vector), *NDUFV3_L_OE* and *NDUFV3_S_OE* HFF measured by CCK-8 assay. (T) SA-β-Gal staining of control and *NDUFV3_L_OE* and *NDUFV3_S_OE* HFF cells. Bars, 200 μm. *, *P* < 0.05; **, *P* < 0.01; ***, *P* < 0.001; ****, *P* < 0.0001, *t*-test. For colour version of this figure refer to online source.

As *PTBP1* knockdown downregulates the exon-preserved transcript (*NDUFV3_L*) and upregulates the exon-skipped transcript (*NDUFV3_S*) of *NDUFV3*, we asked whether decreased *NDUFV3_L* or increased *NDUFV3_S* expression could partially contribute to *PTBP1*-KD induced senescence and related mitochondrial functional changes. We applied three shRNAs that specifically target *NDUFV3_L* (Fig. 5A) to simulate the decreased expression of *NDUFV3_L* upon *PTBP1* knockdown. Consistent with knocking down *PTBP1*, all three shRNAs could downregulate *NDUFV3_L* and responsively upregulate *NDUFV3_S*, that is, the proportion of *NDUFV3_S/NDUFV3* increased significantly (Fig. 5B and 5C). Furthermore, all three shRNAs target to *NDUFV3_L* led to cellular senescence phenotypes, including downregulation of *CDK1*, *MIK67*, upregulation of *CDKN1A* and *CDKN2A*, decreased cell proliferation rate, increased proportion of SA-β-Gal-positive cells and enlarged cell morphology (Fig. 5D–F). Moreover, we found that knockdown of *NDUFV3_L* can also significantly reduce mitochondrial membrane potential (Fig. 5G and 5H) and mildly reduce intracellular ATP concentration (Fig. 5I), while also obviously increase intracellular ROS concentration and mtSOX concentration (Fig. 5J–L). These results indicated that down-regulation of *NDUFV3_L* correlates with *PTBP1*-KD-induced cellular senescence and related mitochondrial functional changes.

Next, we respectively overexpressed *NDUFV3_L* and *NDUFV3_S* in *PTBP1*-KD cells, and we found that over-expression of *NDUFV3_L* but not *NDUFV3_S*, could partially rescue the senescence phenotypes caused by *PTBP1* knockdown (Fig. 5M–Q). These results suggested that decreased expression of *NDUFV3_L* may partially contribute to the phenotypic changes of senescence and mitochondrial functions upon *PTBP1* knockdown. And then, we respectively overexpressed *NDUFV3_L* and *NDUFV3_S* in HFF and HEK293T cells, and found that overexpression of *NDUFV3_S* but not *NDUFV3_L*, could partially induce cellular senescence phenotypes (decreased cell proliferation rate and increased proportion of SA-β-Gal-positive cells) in HFF cells (Fig. 5R–T). Moreover, to a certain extent, overexpression of *NDUFV3_S* could also induce slightly reduced ATP concentration and increased mtSOX concentration in HEK293T cells (Fig. S4), which indicated that increased exon-skipped transcript of *NDUFV3 (NDUFV3_S)* also correlates with and may partially contribute to *PTBP1*-KD induced cellular senescence and related mitochondrial functional changes.

### *PTBP1* expression is negatively correlated with exon skipping of *NDUFV3* in diverse cellular senescence models and cancers

**Figure 6.**
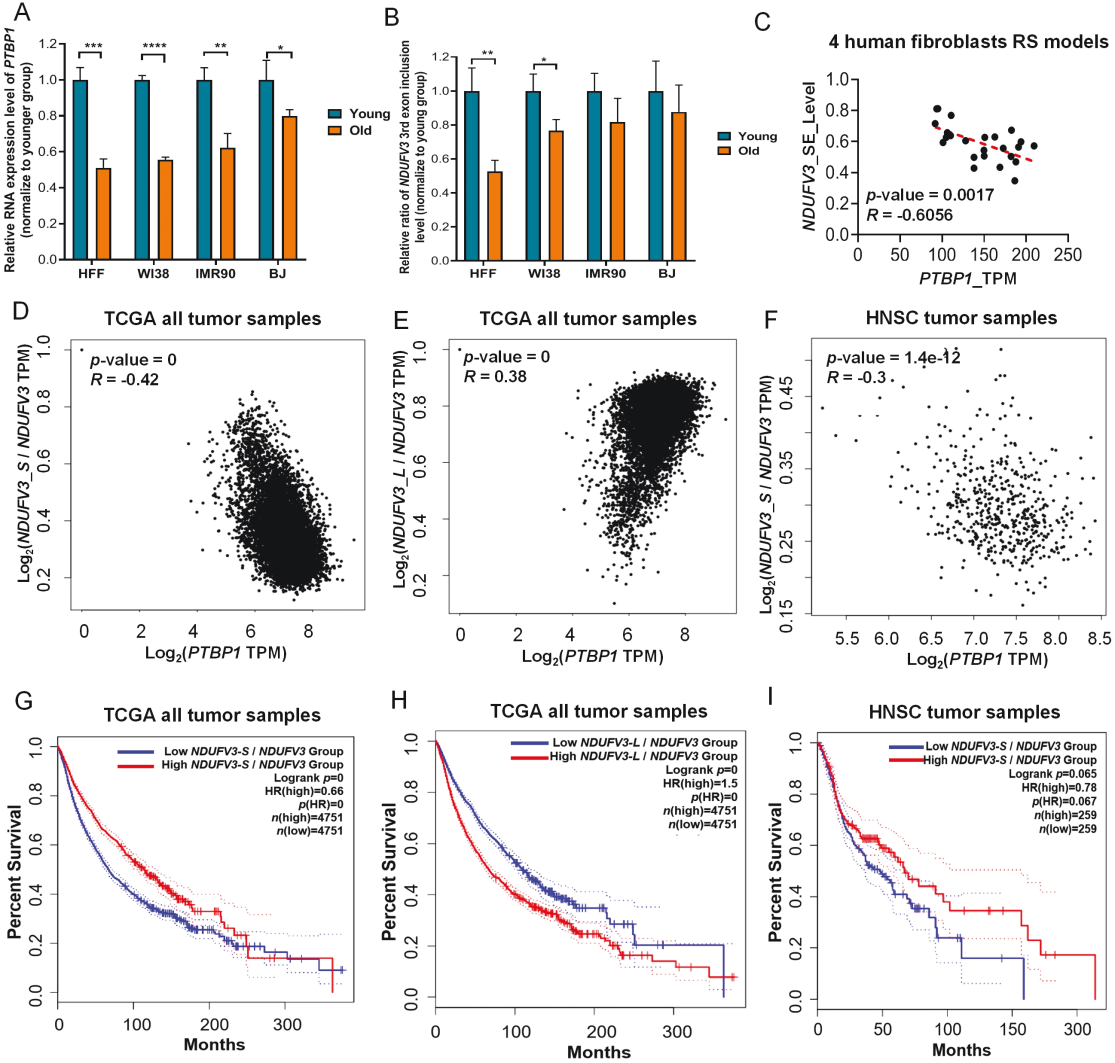
PTBP1 regulates exon skipping of *NDUFV3* in diverse biological processes. (A) Histogram of *PTBP1*’s relative RNA expression level in four replicative senescence models of human fibroblasts (HFF, Human foreskin fibroblasts; WI38, Human embryonic lung fibroblasts, were isolated from the lung tissue of a 3-month-old, female, embryo; IMR90, Human lung fibroblasts, were isolated from normal lung tissue derived from a 16-week-old female; BJ, Human foreskin fibroblasts from a neonatal male). (B) Histogram of the relative ratio of *NDUFV3*’s 3rd exon’s inclusion level in four replicative senescence models of human fibroblasts. (C) Scatter plot and linear correlation analysis of *PTBP1*’s expression level and *NDUFV3*’s exon-skipping level in all 24 samples of four replicative senescence models of human fibroblasts. (D) Scatter plot and linear correlation analysis of *PTBP1*’s expression level and *NDUFV3*’s exon-skipping level (indicated by the proportion of *NDUFV3_S*’s expression level among *NDUFV3*) in all TCGA tumor samples. (E) Scatter plot and linear correlation analysis of *PTBP1*’s expression level and *NDUFV3’*s exon inclusion level (indicated by the proportion of *NDUFV3_L*’s expression level among *NDUFV3*) in all TCGA tumor samples. (F) Scatter plot and linear correlation analysis of *PTBP1*’s expression level and *NDUFV3*’s exon-skipping level in all HNSC (Head and Neck squamous cell carcinoma tumor) samples from TCGA database. (G) Survival analysis of all tumor patients from TCGA database which were divided into two groups based on *NDUFV3*’s exon-skipping level in their tumor samples. (H) Survival analysis of all tumor patients from TCGA database which were divided into two groups based on *NDUFV3*’s exon inclusion level in their tumor samples. (I) Survival analysis of all HNSC tumor patients from TCGA database which were divided into two groups based on *NDUFV3*’s exon-skipping level in their tumor samples. Note: All those correlation analyses above are based on Pearson correlation. *, *P* < 0.05; **, *P* < 0.01; ***, *P* < 0.001; ****, *P* < 0.0001, *t*-test.

To explore whether PTBP1-mediated exon-skipping regulation of *NDUFV3* exists in more biological processes, we used multiple sets of publicly available resources such as Gene Expression Omnibus (GEO), The Cancer Genome Atlas (TCGA), and Gene Expression Profiling Interactive Analysis (GEPIA2) for analysis [36]. In four replicative senescence models of human fibroblasts, we found that *PTBP1*’s relative expression level and *NDUFV3* 3rd exon’s relative inclusion level coordinately downregulated in senescent cells, and *PTBP1* expression level was negatively correlated with the exon-skipping level of *NDUFV3* in these 24 samples (Fig. 6A–C). In TCGA, all tumor samples with diverse human cancer types, a significant negative correlation between *PTBP1* expression and exon skipping of *NDUFV3* was also observed in combined 33 cancer types, and the Head and Neck squamous cell carcinoma (HNSC) serves as a representative example (Fig. 6D–F). In addition, patients with higher exon-skipping degree of *NDUFV3* in their tumor samples not only have better survival rate in combined 33 cancer types but also in given cancer types such as HNSC (Fig. 6G–I). These correlation data support the notion that PTBP1-mediated exon-skipping regulation of *NDUFV3* is prevalent in diverse cellular senescence models and cancers.

## Discussion

**Figure 7.**
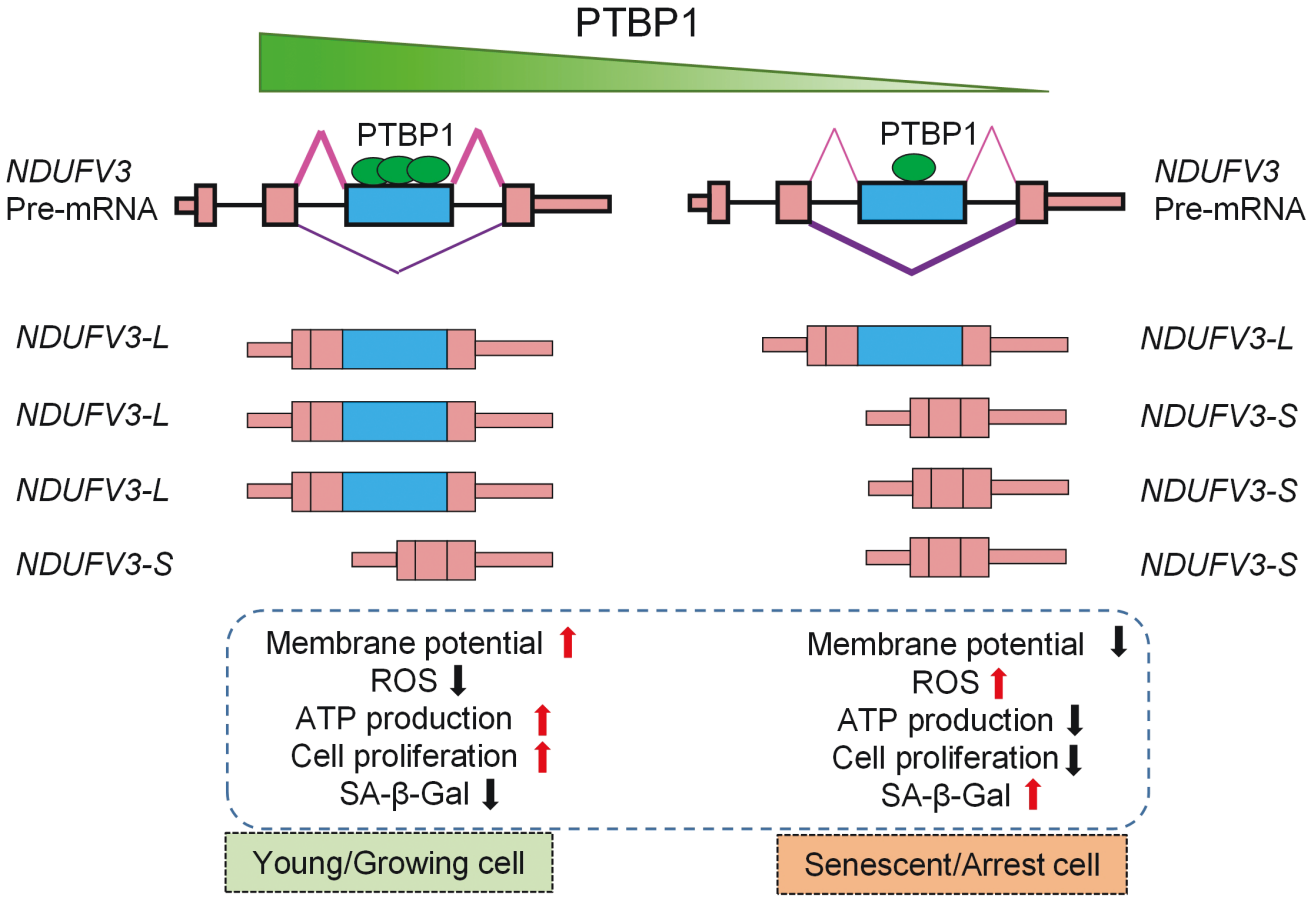
**Working model for *PTBP1*-mediated exon skipping of *NDUFV3* in regulating mitochondrial function and cellular senescence.** *PTBP1* can bind to the 3rd exon region of pre-mRNA transcribed by *NDUFV3* and protect the exon from skipping in younger cells. In senescence cells, the expression level of *PTBP1* decreased, leading to the impaired ability of exon inclusion and therefore promoting exon skipping of exon 3 of *NDUFV3*. The increase of the shorter transcript and reduction of the longer transcript both contribute to the indicated senescence-associated phenotypes and changed mitochondrial functions.

Exon skipping is the most widespread alternative splicing type in animal cells, and previous studies have demonstrated its dynamic changes during cellular senescence [36]. However, the upstream regulatory mechanism and downstream biological effects of exon skipping during cellular senescence remain unclear. In this study, by using HFF replicative cellular senescence as a model, we identified that PTBP1 could act as a key regulator of exon-skipping events during senescence. By knocking down *PTBP1* in HFF cells, we also discovered that downregulation of *PTBP1* could explain 10.6% exon-skipping events changes during HFF replicative cellular senescence, including the exon-skipping event of *NDUFV3*, a well-known mitochondrial complex I subunit gene with two isoforms. This is the first time to reveal one of the core factors that controlling the exon skipping of *NDUFV3*. Functional experiments indicated that knockdown of *PTBP1*-induced cellular senescence and related mitochondrial functional changes correlate with and partially through promoting the exon-skipping event of *NDUFV3* (Fig. 7). We also provide lines of evidence to support that the negative correlation between *PTBP1* and exon skipping of *NDUFV3* exists in other cellular senescence models and diverse cancers, indicating the universality of such splicing regulation mechanism and functional regulatory relationship.

Furthermore, we found that *PTBP1* is not only down-regulated in above mentioned four replicative senescence models of human fibroblasts (Fig. 6A) but also down-regulated in other replicative and inducible cellular senescence models (Fig. S5A), aged tissues and Hutchinson-Gilford Progeria Syndrome (HGPS) patients’ skin-derived fibroblasts (Fig. S5B–E). Given knock down *PTBP1* could induce cellular senescence and related mitochondrial functional changes in this study, we speculated that downregulation of *PTBP1* may act as a prevalent contributor in diverse cellular senescence and individual aging processes and deserve in-depth investigation. Moreover, consistent with the idea that cellular senescence is being regarded as an anti-cancer mechanism and the expression levels of many genes are opposite in senescence and cancer [5, 6], we found that *PTBP1* was up-regulated in most cancer types (Fig. S6A), and its expression level is negatively correlated with patients’ survival rates and positively correlated with the expression levels of such cell proliferation marker genes (such as *CDK1*, *MKI67*, and *LMNB1*, etc.) in all combined cancer types in TCGA (Fig. S6B–E). These results imply that upregulated *PTBP1* may act as a general tumor-promoting factor, and down-regulation of *PTBP1* may also induce cellular senescence phenotypes (like growth arrest) in cancer cells, in line with a study had reported that knock down of *PTBP1* could reduce cell proliferation and invasion abilities in cancer cells [37]. It is also worth noting that in the context of RAS-overexpression and doxorubicin-induced senescence, the authors found that knockdown *PTBP1* could reduce SASP and inhibit inflammation-driven cancer by promoting the exon inclusion of *EXOC7*, and no more cellular senescence phenotypes could be observed (such as cell growth arrest, higher SA-β-galactosidase activity, and upregulation of *CDKN1A* and *CDKN2A*) [38]. In contrast, we found that knocking down *PTBP1* could lead to significant and classical cellular senescence phenotypes such as an increase in the proportion of SA-β-Gal staining positive cells in HFF and HEK293T cells. Two possible reasons to explain the difference. One possible reason is that the RAS-overexpression and doxorubicin treatment they used had already caused the cells to exhibit a very clear and saturated cell senescence phenotype; therefore, knocking down *PTBP1* can no longer exhibit any more classical senescence phenotype. In contrast, we performed such studies in untreated cells. The other possible reason is the cell type difference. To reveal and understand *PTBP1*’s cellular functions in different cell types and biological progresses, further investigations are required.

In this study, we also identified *NDUFV3* as a novel gene to regulate senescence. No evidence indicates the casual relationship between *NDUFV3* and cellular senescence. We found that not only specifically knocking down *NDUFV3_L* (Fig. 5) but also knocking down *NDUFV3* itself could lead to senescence-associated phenotypes and related mitochondrial functional changes (Fig. S7). These results indicated that NDUFV3 is an important subunit of mitochondrial complex I, and the differential use of the long and short transcripts of *NDUFV3* mediated by PTBP1’s expression change may have important regulatory effects on the mitochondrial and cellular functions of tissue cells in various biological processes. These conclusions maybe different to some extent from some published works by Michael Ryan and colleagues, which reported that cell lines lacking NDUFV3 could still grow on galactose with negligible to moderate reductions in complex I activity and mitochondrial respiratory capacity, and indicated that NDUFV3_L and NDUFV3_S could be all successfully participated in the assembly of complex I, these results seem implied that expression change of *NDUFV3* and the differential use of its two isoforms may not play important roles in biological processes [39, 40]. We think there were two possible reasons to explain the differences between their and our studies. First, their studies and our studies used different cell types. Michael Ryan’s researches were performed in HEK293T cells [39, 40], while our study was carried out mainly in primary HFF, NDUFV3 may show varying roles on mitochondrial and cellular functions between primary and immortal cells. Second, the two works applied different gene knockdown/knockout methods. We used shRNA to knockdown *NDUFV3* and *NDUFV3_L*, while they used TALEN and CRISPR/Cas9 gene-editing tools to disrupt related genes [39, 40]. Jun Chen’s and Didier Y R Stainier’s studies have reported that gene mutations but not RNA interference may lead to premature-stop codon and, therefore, nonsense-mediated decay (NMD) in related mRNA, and in turn, trigger genetic compensation [41, 42], thus preventing the present of the true phenotype of the gene deletion. The distinct methods used for silencing the gene *NDUFV3* may account for the inconsistency. Moreover, we also designed multiple shRNAs to target different regions of *NDUFV3* to minimize the impact of off-target effects on the conclusion (Fig. 5A), and a recent study had reported that heart with higher ratio of *NDUFV3-S*/*NDUFV3-L* could cause mitochondria to produce more ROS to injure tissues and cells during ischemia/reperfusion (I/R) [43], which may provide an evidence to support for our conclusions. So, we supposed that *NDUFV3* and the different uses of its two isoforms may play important roles in cellular senescence, regulation of mitochondrial functions, and other complex biological processes. Interestingly, *NDUFV3* is few of the mitochondrial-relevant genes that could be regulated by exon skipping. We, therefore, firstly discovered an example that exon skipping of mitochondrial-related gene could simultaneously regulate and link cellular senescence and mitochondrial dysfunction, expanding the functional knowledge regarding mitochondrial genes and senescence.

## Research limitations

In this study, even we firstly found that *PTBP1* downregulation could induce cellular senescence and related mitochondrial functional changes partly via promoting exon skipping of *NDUFV3* in HFF and HEK293T cells, and correlation analysis based on published datasets indicated that such splicing regulation mechanism may generally exist in diverse biological progresses. However, such evidence has been derived from *in vitro* systems and online datasets. Thus, validating the changing patterns and functions of PTBP1 and its regulated exon-skipping events of *NDUFV3* in an *in vivo* model is desirable in the future. In addition, due to this study’s focus on cellular senescence, even we indicated that such splicing regulation mechanism may also generally exist in diverse cancers but we have not performed functional experiment in cancer cell lines in this study; these will be carried out in our next study. Moreover, some potential clinical applications are worthy of further study. Furthermore, even though there were over a hundred differential exon-skipping events related genes are mitochondrial localization protein genes but by comprehensively considering whether such differential exon-skipping events in HFF replicative senescence were potentially directly regulated by PTBP1, the significance of exon-skipping level changes upon *PTBP1* knockdown (PSI ≥ 0.2, *P* value ≤ 0.05) and consistent in HFF and HEK293T cells, related gene expression levels (mean transcripts per million (TPM) ≥ 10), main transcripts numbers (≤ 3), and the potential importance of corresponding gene functions. We ultimately selected *NDUFV3* as a candidate gene that meets the above criteria for downstream functional and mechanistic studies, but it is likely that other mitochondrial-related gene expression and alternative splicing changes could also play important roles in cellular senescence and related mitochondrial functional changes, which need further investigation. Finally, more experimental methods could be used for the future study. For example, to more completely measure the energy status of the cell, the phosphorylation potential [ATP]/([ADP][Pi]) could be carried out.

## Methods

### Materials

#### Cells and plasmids

Primary HFF cells were provided by Cell Bank, Chinese Academy of Sciences. HEK293T cells were provided by ATCC. Plasmids, including pLKO.1, psPAX2 packaging plasmid, pMD2.G envelope plasmid, were provided by addgene. PCDH-CMV-CMS-EF1A-BSD plasmid were modified by combining the PCR products of PCDH-CMV-CMS-EF1A-copGFP-T2A-BSD and lentiCas9-Blast plasmid, which were provided by professor YongMing Wang from Fudan university.

#### Public RNA-seq and eCLIP-seq datasets

The RNA-seq dataset of four human cellular senescence models (including HFF, MRC-5, BJ, IMR90, and WI38) is available on Gene Expression Omnibus [GEO] under accession number GSE63577. The RNA-seq datasets of other replicative cellular senescence (RS) and inducible cellular senescence (IS) are also available on GEO (accession number of HDF-RS’s RNA-seq data is GSE210020; accession number of ESLFC-RS’s RNA-seq data is GSE109700; accession number of BMMSC-RS’s RNA-seq data is GSE178514; accession number of IMR90-H2O2-IS’s RNA-seq data is GSE134088; accession number of astrocytes-oxdative-IS’s RNA-seq data is GSE58910; accession number of IMR90-bleomycin-IS’s RNA-seq data is GSE168994; accession number of WI38-doxprubicin-IS’s RNA-seq data is GSE130727; accession number of ionizing radiation exposure (IR) inducible senescence models’ RNA-seq data in WI38, HUVEC, HAEC cells is GSE130727). The RNA-seq dataset of 133 healthy individuals’ and 10 HGPS patients’ skin tissues driven dermal fibroblasts of different ages is available on GEO under accession number GSE113957. The RNA-seq dataset of 86 human donor lungs is available on GEO under accession number GSE165192. The RNA-seq dataset of PTBP1/2 knock down in HEK293T cells is available on GEO under accession number GSE69656. The eCLIP-seq datasets of PTBP1 and other RBPs were obtained from POSTAR 3.0 and ENCODE (Encyclopedia of DNA Elements).

### Methods

#### HFF and HEK293T cells culture and passage

Cells were cultured in DMEM (Gibco) supplemented with 15% (HFF cells) and 10% (HEK293T cells) FBS (Gibco) at 37°C in a humidified, 5% CO_2_ incubator. When the cell confluence reached about 95%, the cells were sub-cultured at 1:4.

#### Lentiviral package in HEK293T cells and transfection to HFF and HEK293T cells

For stable knockdown (or over-expression) of target genes (or transcripts), we applied a lentivirus transfection-mediated gene-silencing (or gene over-expressing) strategy. Oligos of shRNAs (shRNA target sequences are listed in Table S2) were annealed and then cloned into pLKO.1 vector (over-expressed transcripts fragments were amplified from human cDNA and were cloned into PCDH-CMV-CMS-EF1A-BSD vector; the sequences were overexpressed in this study of *NDUFV3_L* and *NDUFV3_S* are listed in Tables S4 and S5). HEK293T cells grown in six-well plates were transfected with 1 μg constructed vectors or negative control vectors (including pLKO.1 and PCDH-CMV-CMS-EF1A-BSD) with 0.75 μg psPAX2 packaging plasmid and 0.25 μg pMD2.G envelope plasmid using Lipofectamine 2000 (Invitrogen). After culturing for 36–48 h, the virus supernatant was harvested and filtered by 0.45 μm millipore filter membrane and then to infect HFF or HEK293T cells in six-well plates. After incubation over 36 h, infected HFF and HEK293T cells were screened by 2.5 μg/mL puromycin (Sigma, 540222) or 10 μg/mL Blasticidin (Solarbio, B9300) for 2 days. The surviving cells were sub-cultured and cultured for two more days and then used for cell proliferation assay, RNA extraction, and other functional experiments.

#### RNA extraction, RT-PCR, and qRT-PCR

Total RNA was isolated with TRIzol reagent according to manufacturer’s instructions (Invitrogen, 15596026). Then, RNA was reversely transcribed into cDNA with oligo-(dT)_23_ VN primer and (or) random hexamers using HiScript II 1st Strand cDNA Synthesis Kit (+gDNA wiper) (Vazyme, R212). Gene expression at the RNA level was quantified by qRT-PCR using 2× SYBR mix (Vazyme, Q711). *GAPDH*’s RNA expression level served as an internal control. Then, the reaction was run on Bio-Rad CFX96 manager machine. Primer sequences are listed in Table S3.

#### Cell proliferation assay

Cell proliferation assay was performed on cultured cells at four time points (24, 48, 72, and 96 h for HEK293T cells, and for HFF cells, will be appropriately prolonged). Cells were counted and seeded in 96-well plates with 1000 cells per well and three replicates for each time points. Cell Counting Kit-8 (CCK-8) reagent (Dojindo, CK04) was diluted with DMEM according to the manufacturer’s protocol and then added to each testing well. Then, cells were incubated at 37°C for another 3 h, and then the absorbance of each well was measured at 450 nm by a microplate reader (SPARK), and then we normalized the absorbance of the third or fourth time point to the absorbance of the first time point, as the relative cell proliferation rate.

#### Senescence-associated β-galactosidase staining

Senescence Cells Histochemical Staining Kit (Sigma, CS0030-1 KT) was used for SA-β-Gal activity detection. Cells were seeded in 48-well plates at about 70% confluence. After a certain period (about one day or more) of cell culture (excessive density needed to be avoided), we removed the DMEM medium; cells were washed twice with 1× PBS and incubated for 6–7 min with 0.6 mL 1× fixation buffer per well. After fixation, cells were washed twice with 1× PBS. The staining solution mixture (10× staining solution, reagent B, reagent C, X-gal solution, and ddH_2_O, prepared according to the manufacturer’s protocol) was added to cells. Cell culture plates were sealed with sealing film to isolate air and incubated at 37°C for several hours (about 6–8 h for HFF cells and 14–16 h for HEK293T cells) and then observed under an inverted microscope (Olympus X73 and Leica).

#### Mitochondrial membrane potential detection

JC-1 MitoMP Detection Kit (Dojindo, MT09) was used for mitochondrial membrane potential detection in HEK293T cells and HFF cells. About 2000 cells (HFF cells) or 5000 cells (HEK293T) were each seeded in those pores of 96-well plate; after a certain period (about one day or more) of cell culture (excessive density needed to be avoided), we removed the DMEM medium, and added 100 μL prepared JC-1 working solution (2 μM JC-1 dye in DMEM medium with 10% FBS), and then incubated for 30 min in a 37°C 5% CO_2_ incubator. After incubation, the working solution was removed and washed twice with HBSS, and added 100 μL Imaging Buffer Solution (1×), then observed under an inverted fluorescent microscope (Olympus X73) or measured by Microplate reader (SPARK) with corresponding fluorescence parameter (Green: Ex = 488 nm, Em = 500–550 nm; Red: Ex = 561 nm, Em = 560–610 nm).

#### Detection of total intracellular ATP concentration

Since the infection efficiency of HFF cells is too low to obtain enough cells, we used HEK293T to study the influence of such treatments on total intracellular ATP concentration. We used a kit named Enhanced ATP Assay Kit (Beyotime, S0027) to detect the total intracellular ATP concentration of HEK293T cells, and using protein concentration valued by an Omni_EasyTM instant BCA Protein Assay Kit (epizyme, ZJ102) as the internal reference among samples. About 20,000 cells for each sample were seeded in each pore of 24-well plates. One day later, the DMEM medium was removed and washed one time with PBS, and then added 200 μL ATP detection lysate to lyse cells. After centrifuge at 4°C 12,000 *g* for 5 min, 20 μL of cell lysate was aspirated from each sample (three replicates totaling 60 μL) for total ATP detection, 20 μL of cell lysate was aspirated from each sample (three replicates totaling 60 μL) for protein concentration detection. Follow-up experimental operations were conducted according to the instructions and measured by a Microplate reader (SPARK).

#### Co-detection of intracellular ROS and mtSOX levels

We used a kit named ROS Assay Kit-Highly Sensitive DCFH-DA (Dojindo, R253) to detect the total intracellular ROS in HEK293T and HFF cells, and intracellular mitochondrial superoxide level was detected at the same time by a kit named mtSOX Deep Red-Mitochondrial Superoxide Detection (Dojindo, MT14), and hoechst 33342 (C1022, Beyotime) was used to stain chromatin at the same time and the fluorescence value (Blue: Ex = 350 nm, Em = 461 nm) as the internal reference among samples. About 2000 cells (HFF cells) or 5000 cells (HEK293T cells) of each sample were seeded in each pore of 96-well plates (Black transparent bottom, FCP965-8pcs, Beyotime). We then carried out the experiment according to the instructions (Three dyes were dissolved together in the culture medium according to their corresponding working concentrations, thus achieving co staining), and then observed and photographed under an inverted fluorescent microscope (Olympus X73) with corresponding fluorescence parameter (Blue for chromatin: Ex = 350 nm, Em = 461 nm; Green for intracellular ROS: Ex = 488 nm, Em = 510–535 nm; Red or deep-red for intracellular mitochondrial superoxide: Ex = 535–565 nm, Em = 640–700 nm), and maintain consistency of microscope parameters when observing different samples.

#### Process of published RNA-seq data

RNA-seq reads were mapped to human genome hg38 (GENCODE V40) using STAR [44], and the uniquely mapped reads were kept for further analysis. The gene expression level for each sample specified as TPM was calculated by Salmon [45]. Gene expression changes between two conditions were estimated using DESeq2 R package [46]. rMATS-turbo [24] was used to analyze differential AS events between senescent and young HFF and other fibroblast cells, with inclusion level difference > 10% and FDR < 0.05.

#### Oxford Nanopore technologies (ONT) based long read RNA sequencing and analysis for poly(A)^+^ selected RNA

After RNA extraction, the RNA samples were sent to a company based on Oxford nanopore technologies (ONT) to perform the poly(A)^+^ RNA selection, libraries construction, and Nanopore-based long-read RNA sequencing. Then, we performed bioinformatics analysis for Nanopore sequencing data as following. Low-quality sequencing data is first filtered out, and the presence of primers at both ends of the sequencing sequence is used to determine whether it is a full-length sequence. Perform the polish step on the full-length sequence to obtain a consistent sequence. Merge the consistency sequence of each sample, using Minimap2 [47], which aligns consistent sequences, compares the results to remove redundancy, filters out sequences with consistency less than 0.9 and coverage less than 0.85, and merges alignments with differences only at the 5ʹ end. Finally, 27,325 non-redundant transcript sequences were obtained, and these transcript sequences could be used for alternative splicing analysis.

The differential expression and splicing of Nanopore are based on the FLAIR analysis process [48]. Analysis of gene differential expression using DESeq2 packaged with FLAIR [46], corrected *P* value < 0.05, and log_2_ (FoldChange) ≥ 1 indicates differential expression of the gene. Analysis of differential splicing events using FLAIR packaged DRIMSeq [34]. A corrected *P* value of < 0.05 and an absolute difference in the expression values of selective splicing between groups of > 0.1.

#### Images drawing and fluorescent images processing and analysis

Wiggle plots of such genes and transcripts based on processed sequencing data were drawn by Integrative Genomics Viewer (IGV) [49]. Other images such as qRT-PCR results were drawn by Graphpad Prism 8.0. Fluorescent images are merged and analyzed by ImageJ.

#### Online analysis by Gene Expression Profiling Interactive Analysis (GEPIA2)

Scatter plots, linear correlation analysis, and patients’ survival rate analysis in diverse tumor samples were performed by online tools of GEPIA2 [36].

#### Statistical analysis

In this work, *t*-test was used for all those comparative results, such as genes and transcripts expression levels by qPCR and RNA-seq, intracellular ATP concentration, intracellular ROS and mtSOX concentration indicated by relative fluorescence intensity, and so on, and asterisk (*) represent statistical significance (*P* < 0.05); **, *P* < 0.01; ***, *P* < 0.001; ****, *P* < 0.0001. Moreover, Pearson Correlation Analysis and *t*-test were used for all those correlation analyses, and the log-rank test was chosen on GEPIA2 for all those survival analyses.

### Research ethics

This study mainly conducted in commercially purchased human primary foreskin fibroblasts (HFF) and HEK293T cells and did not involve human stem cells, the human body, or animal experiments. Therefore, this study did not involve corresponding research ethical issues, and thus, it met the ethical requirements for research.

## Supplementary Material

lnae021_suppl_Supplementary_Material

## Data Availability

Raw data of RNA-seq and Oxford Nanopore Technologies (ONT) based long read RNA sequencing of poly(A)^+^ selected RNA with and without *PTBP1* knockdown in HFF cells can be found at NCBI Sequence Read Archive (SRA) under accession number PRJNA1105378, and those raw data of RNA-seq can also be found at NCBI Gene Expression Omnibus (GEO) under accession number GSE266081. Other data supporting the findings of this study are available within the article and its supplementary materials.
