# Supplementary material for: Cellular senescence induced by down-regulation of PTBP1 correlates with exon skipping of mitochondrial-related gene NDUFV3
Source: Life Med. 2024 May 3;3(2):lnae021. doi: 10.1093/lifemedi/lnae021 (PMC11749709; doi:10.1093/lifemedi/lnae021)
Supplement: lnae021_suppl_Supplementary_Material [file lnae021_suppl_Supplementary_Material.docx]

**Cellular senescence induced by down-regulation of *PTBP1* correlates with exon skipping of mitochondrial related gene *NDUFV3***

Yu Yang^1,2,†^, Haimei Wen^2,†^, Yuxin Li^2^, Xin Zeng^2^, Gang Wei^2,3^, Zhenglong Gu^1,2*^, Ting Ni^2,4,5,*^

^1^Center for Mitochondrial Genetics and Health Research, Greater Bay Area Institute of Precision Medicine (Guangzhou), Fudan University, Guangzhou 511400, China

^2^Collaborative Innovation Center of Genetics and Development, Human Phenome Institute, School of Life Sciences, Fudan University, Shanghai 200438, China

^3^MOE Key Laboratory of Contemporary Anthropology, School of Life Sciences, Fudan University, Shanghai 200438, China

^4^National Clinical Research Center for Aging and Medicine, Huashan Hospital, Fudan University, Shanghai 200438, China

^5^State Key Laboratory of Reproductive Regulation and Breeding of Grassland Livestock, Institutes of Biomedical Sciences, School of Life Sciences, Inner Mongolia University, Hohhot 010070, China

^†^These authors contributed equally to this work.

^*^Correspondence: guzhenglong@ipm-gba.org.cn (Z.G.), tingni@fudan.edu.cn (T.N.)

## Supplemental Figures


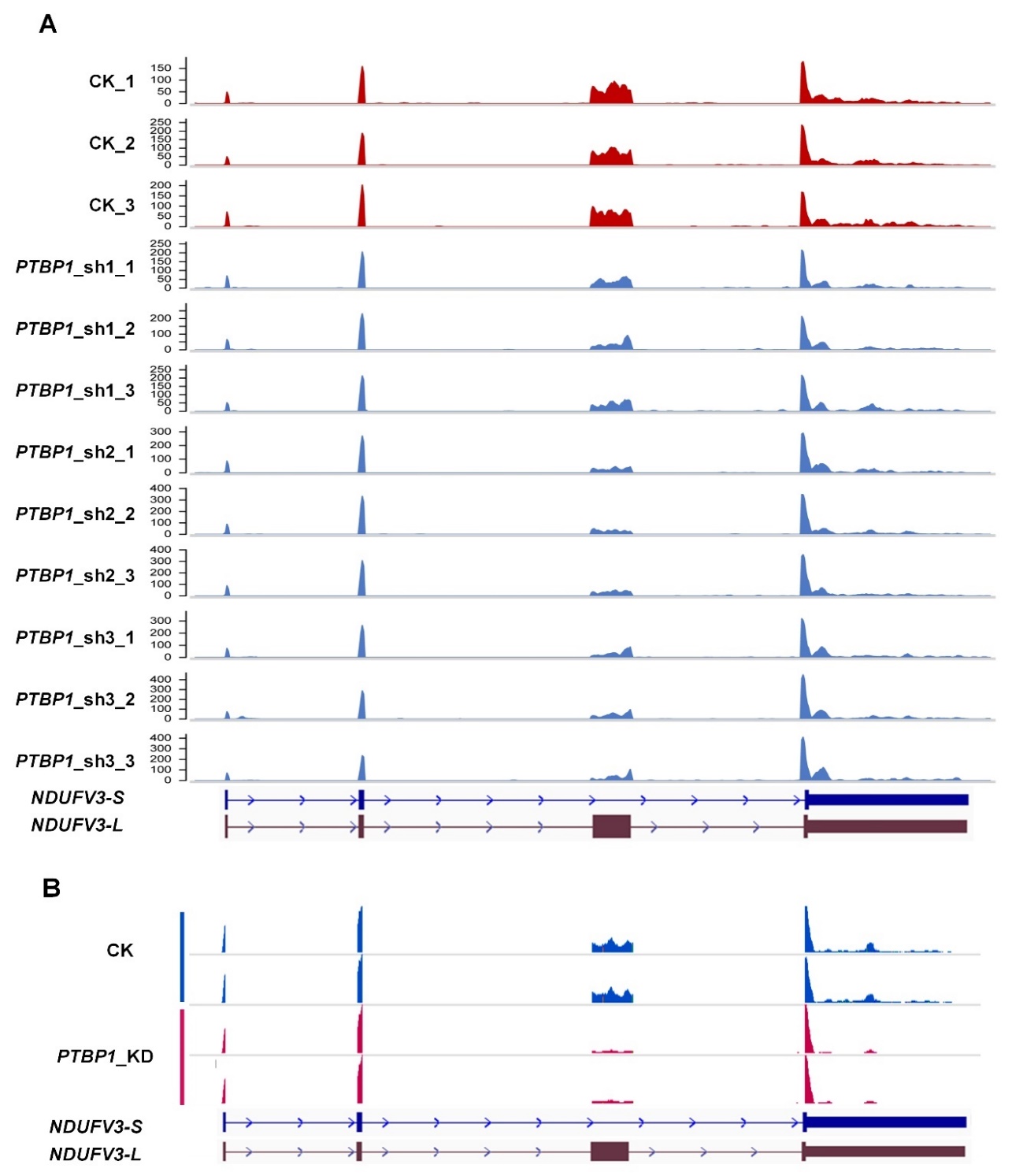


**Figure S1. Wiggle plots by poly(A)+ RNA-seq data showing that knockdown of *PTBP1* could leads upregulated exon skipping of *NDUFV3* in HFF and HEK293T cells.**

(A) Wiggle plots by poly(A)+ RNA-seq data showing that knockdown of *PTBP1* could leads upregulated exon skipping of *NDUFV3* in HFF. (B) Wiggle plots by published poly(A)+ RNA-seq data (GEO: GSE69656) showing that knockdown of *PTBP1* could leads upregulated exon skipping of *NDUFV3* in HEK293T cells.


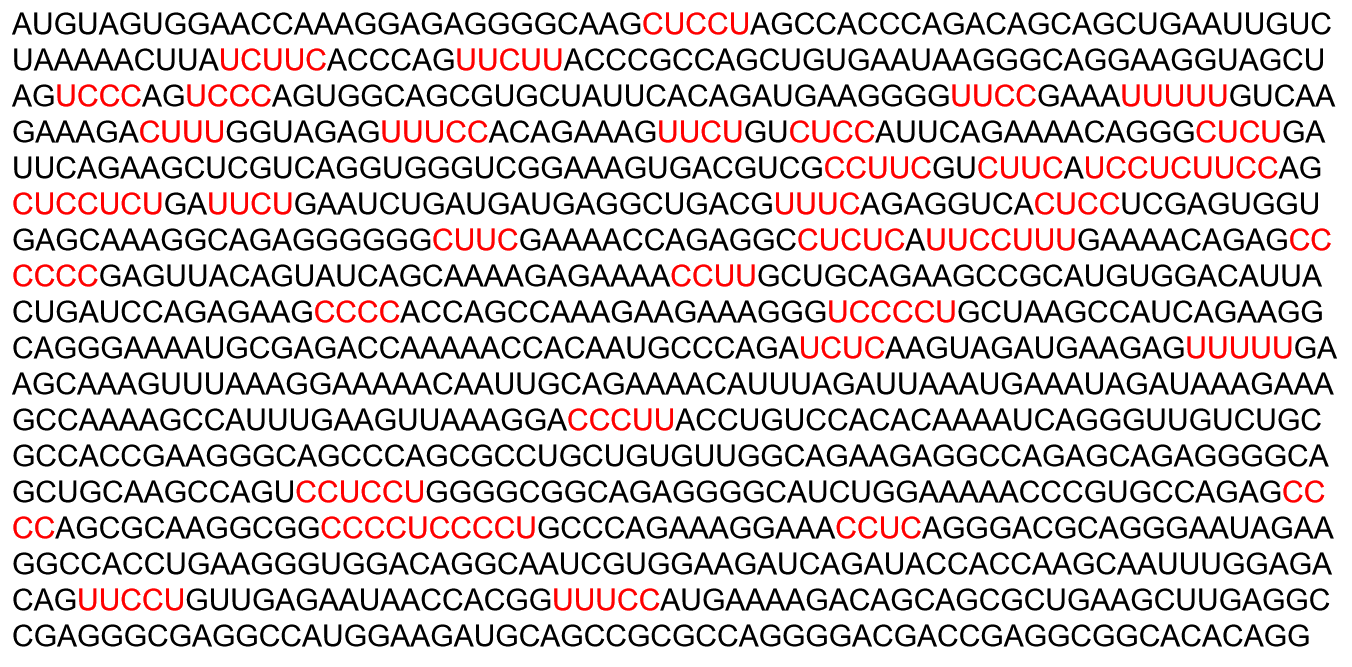


**Figure S2. Potential binding motif sequences (marked red) of PTBP1 on the 3rd exon of *NDUFV3*’s pre-mRNA.**


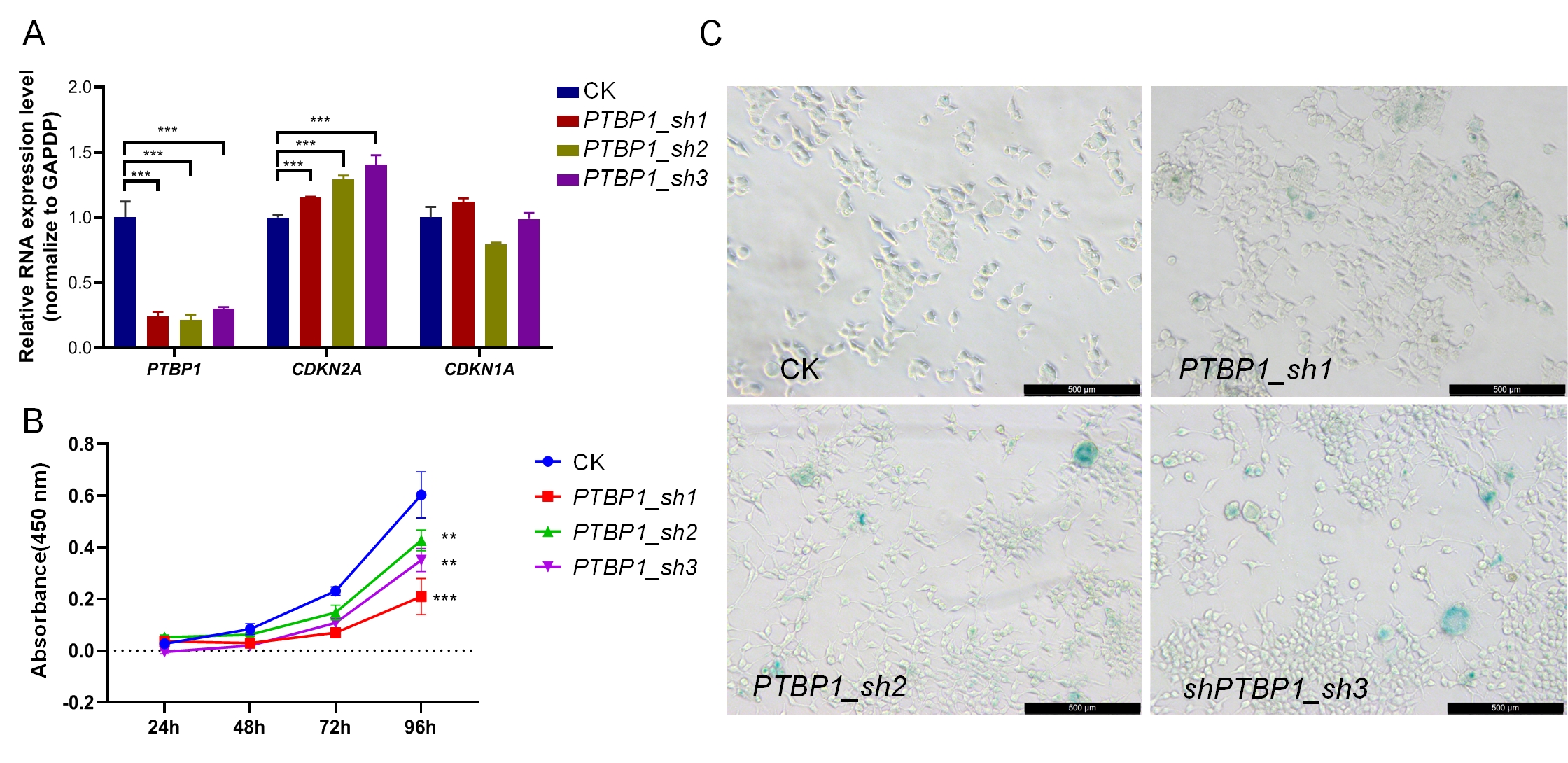


**Figure S3. Knockdown of *PTBP1* leads to cellular senescence in HEK293T cells.**

(A) Gene expression evaluation of *PTBP1, CDKN2B* and *CDKN1A* before and after *PTBP1* knockdown in HEK293T cells by qRT-PCR. (B) Proliferation rate of control (CK) and *PTBP1*_KD HEK293T cells measured by CCK-8 assay. (C) SA-β-Gal staining of control (CK) and *PTBP1*_KD HEK293T cells. Bars, 500 μm. *, *p* < 0.05; **, *p* < 0.01; ***, *p* < 0.001; ****, *p* < 0.0001, *t*-test.


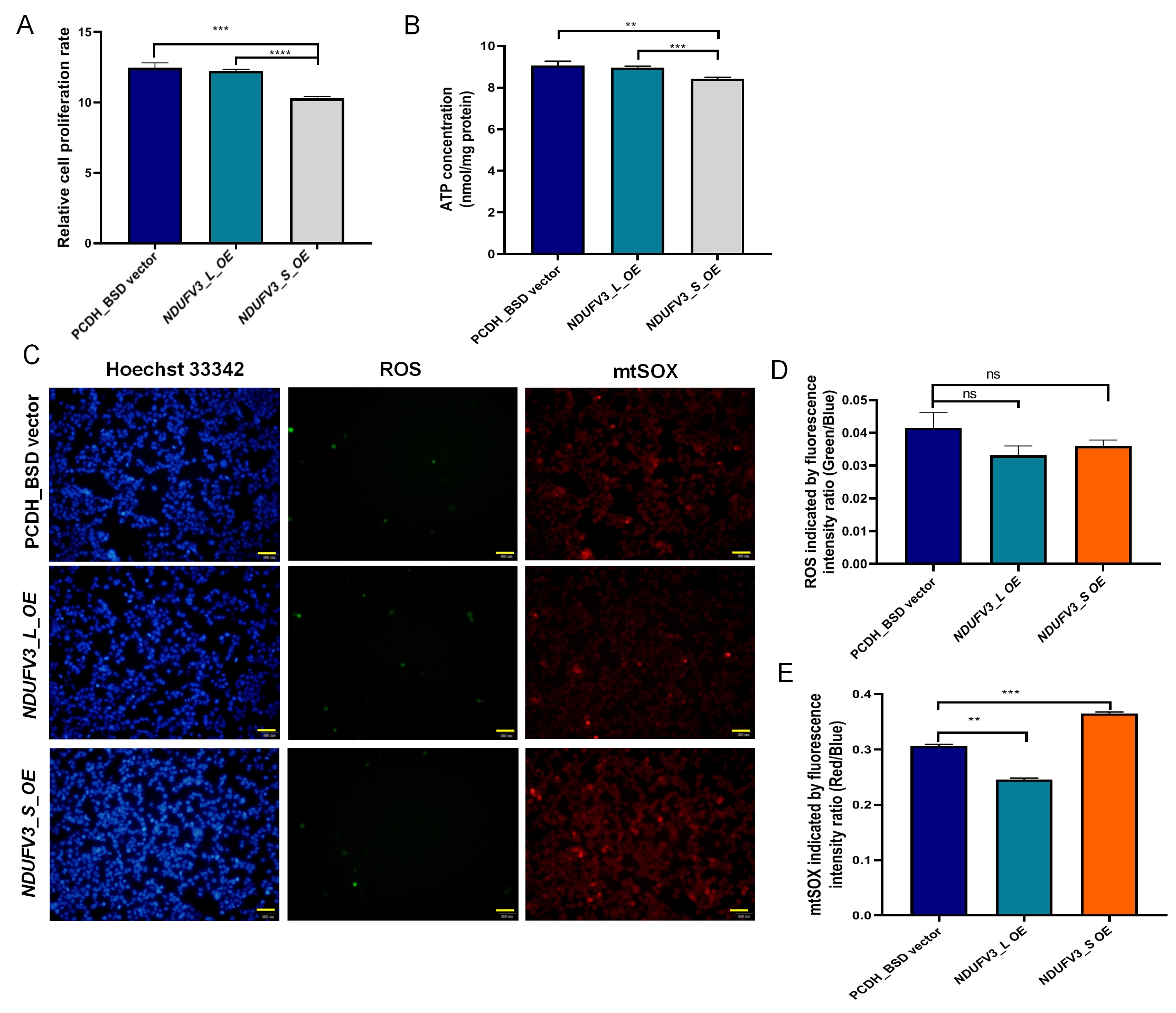


**Figure S4. Overexpression of *NDUFV3_S* but not *NDUFV3_L* could also induce related mitochondrial functional change.**

(A) Relative proliferation rate of control (PCDH_BSD vector), *NDUFV3_L* overexpressed and *NDUFV3_S* overexpressed HEK293T cells measured by CCK-8 assay. (B) ATP concentration in control (CK) and *NDUFV3_L* and *NDUFV3_S* overexpressed HEK293T cells. (C) Fluorescence staining images of chromatin (Hoechst 33342), intracellular reactive oxygen species (ROS) and mitochondrial superoxide (mtSOX) in control (PCDH_BSD vector), *NDUFV3_L*_*OE* and *NDUFV3_S_OE* HEK293T cells. Bars, 500 μm. (D) Quantitative measure of intracellular reactive oxygen species (ROS) relative amount base on fluorescence intensity ratio (Green/Blue) in control (CK) and *NDUFV3_L* and *NDUFV3_S* overexpressed HEK293T cells. (E) Quantitative measure of intracellular mitochondrial superoxide (mtSOX) relative amount base on fluorescence intensity ratio (Red/Blue) in control (CK) and *NDUFV3_L* and *NDUFV3_S* overexpressed HEK293T cells.*, *p* < 0.05; **, *p* < 0.01; ***, *p* < 0.001; ****, *p* < 0.0001, *t*-test.


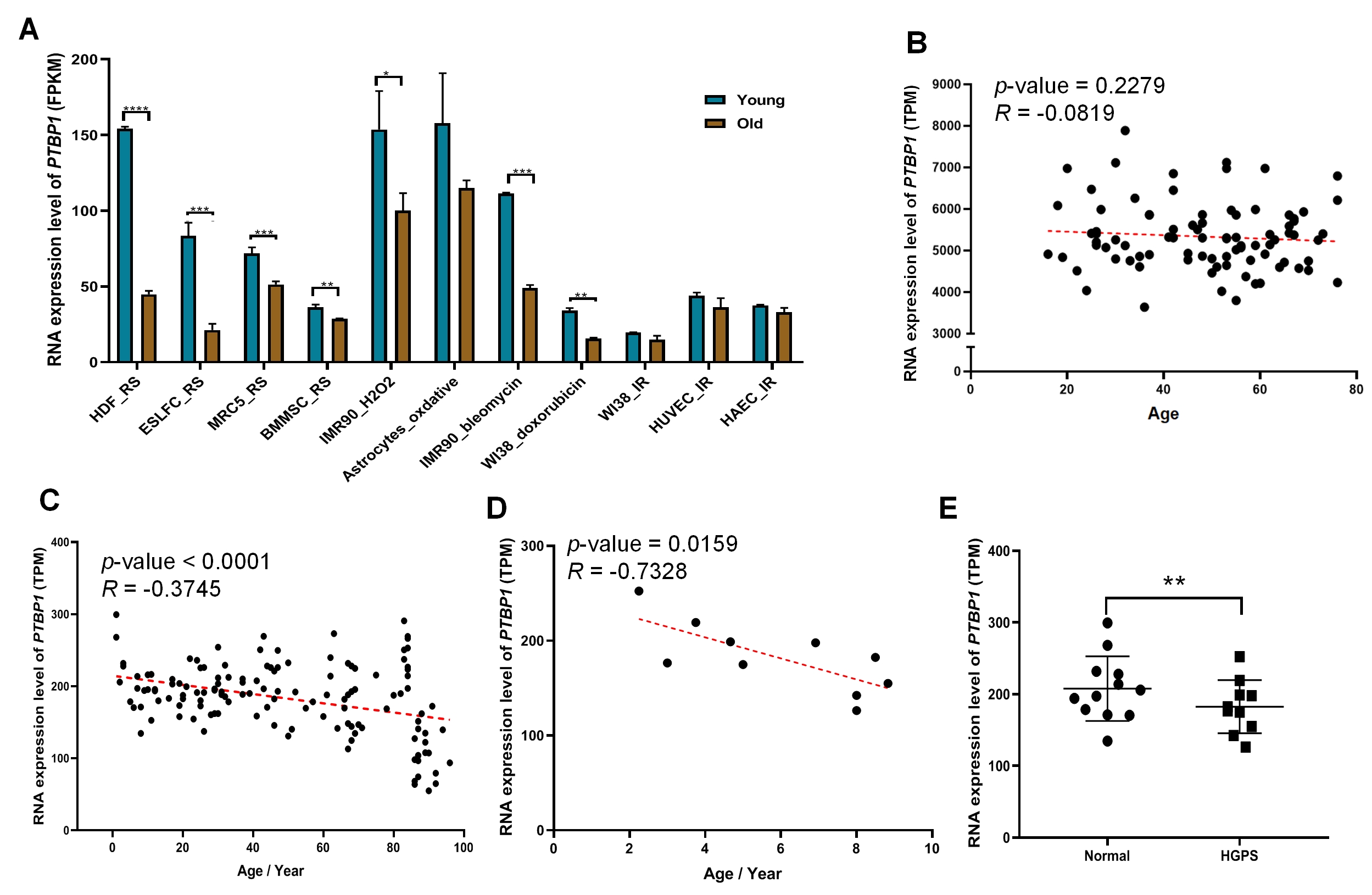


**Figure S5. *PTBP1* is downregulated with various cellular senescence models and aging processes of tissues.**

(A) Histogram of *PTBP1*’s RNA expression level in other four replicative senescence models and seven inducible cellular senescence models (RS means replicative senescence; IR means Ionizing radiation exposure; HDF, human dermal fibroblasts; ESLFC, ES-derived lung fibroblast cells; MRC5, human embryonic lung fibroblasts; BMMSC, bone marrow-derived mesenchymal stem cell; HUVEC, human umbilical vein endothelial cells; HAEC, human aortic endothelial cells; IMR90, human lung fibroblasts; WI38, human embryonic lung fibroblasts). *, *p* < 0.05; **, *p* < 0.01; ***, *p* < 0.001; ****, *p* < 0.0001, *t*-test. (B) Scatter plot and linear correlation analysis of age and *PTBP1’s* expression level in 86 human donor lungs with age ranging from 16 to 76 years (GEO data base accession number is GSE165192). (C) Scatter plot and linear correlation analysis of age and *PTBP1*’s expression level in human dermal fibroblasts driven from 133 healthy individuals’ skin with age ranging from 1 to 94 years (GEO database accession number is GSE113957). (D) Scatter plot and linear correlation analysis of age and *PTBP1*’s expression level in human dermal fibroblasts driven from skin tissues of 10 Hutchinson-Gilford Progeria Syndrome (HGPS) patients with age ranging from 1 to 9 years (GEO database accession number is GSE113957). (E) Scatter plot of *PTBP1*’s RNA expression level in human dermal fibroblasts driven from 12 healthy individuals’ and 10 HGPS patients’ skin tissues with age ranging from 1 to 9 years (GEO database accession number is GSE113957). **, *p* < 0.01; wilcoxon signed rank test.


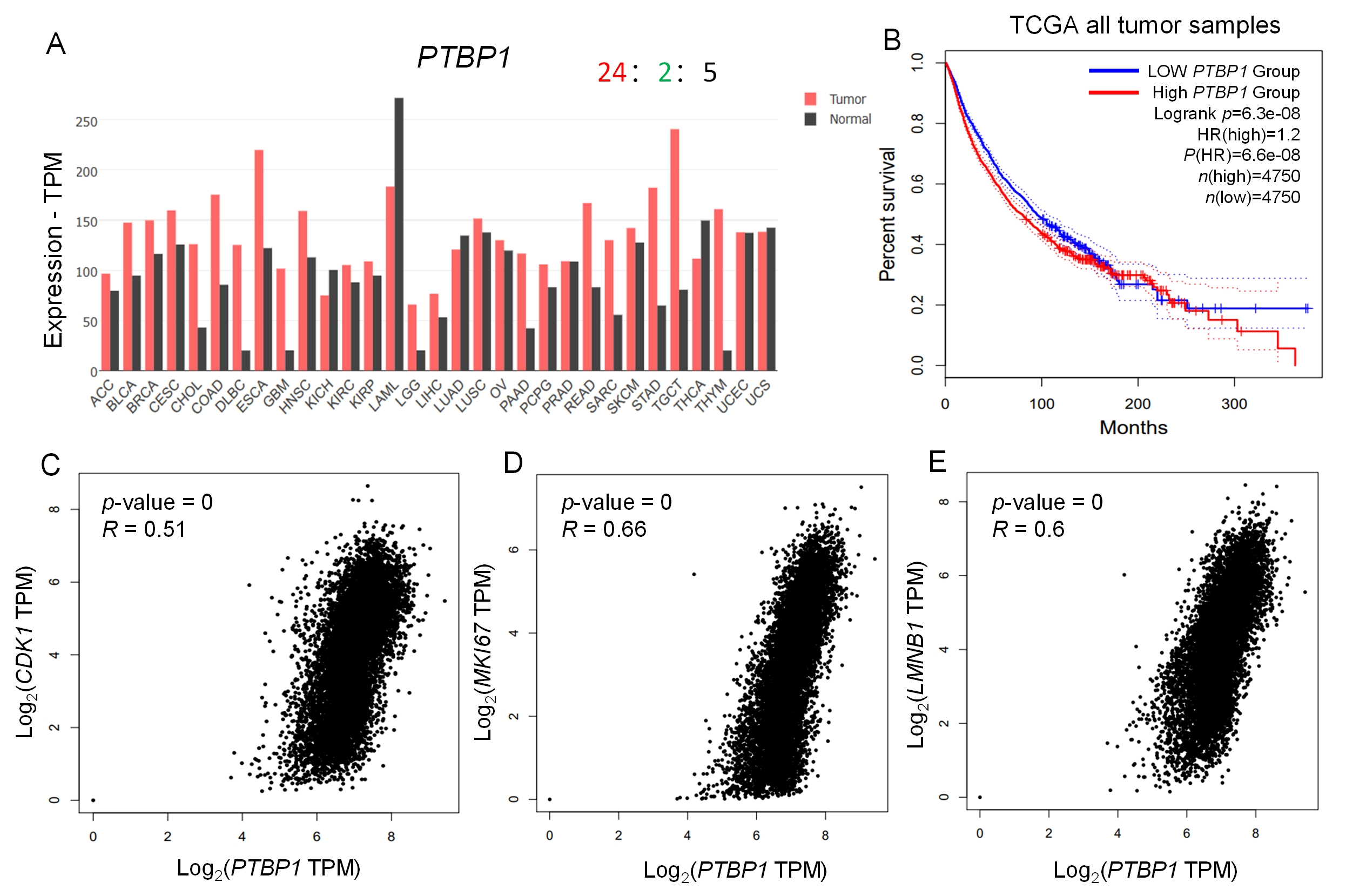


**Figure S6. PTBP1 may be a general** **tumor-promoting factor.**

(A) Histogram of *PTBP1*’s RNA expression level in 31 types’ tumor samples and paired normal samples. The number indicate that *PTBP1* is upregulated in 24, unchanged in 2, and downregulated in 5 types’ tumor. (B) Survival analysis of all tumor patients from TCGA database which were divided into two groups based on *PTBP1*’s expression level in their tumor samples. (C) Scatter plot and linear correlation analysis of *PTBP1*’s expression level and *CDK1*’s expression level in all TCGA tumor samples. (D) Scatter plot and linear correlation analysis of *PTBP1*’s expression level and *MKI67*’s expression level in all TCGA tumor samples. (E) Scatter plot and linear correlation analysis of *PTBP1*’s expression level and *LMNB1’s* expression level in all TCGA tumor samples.


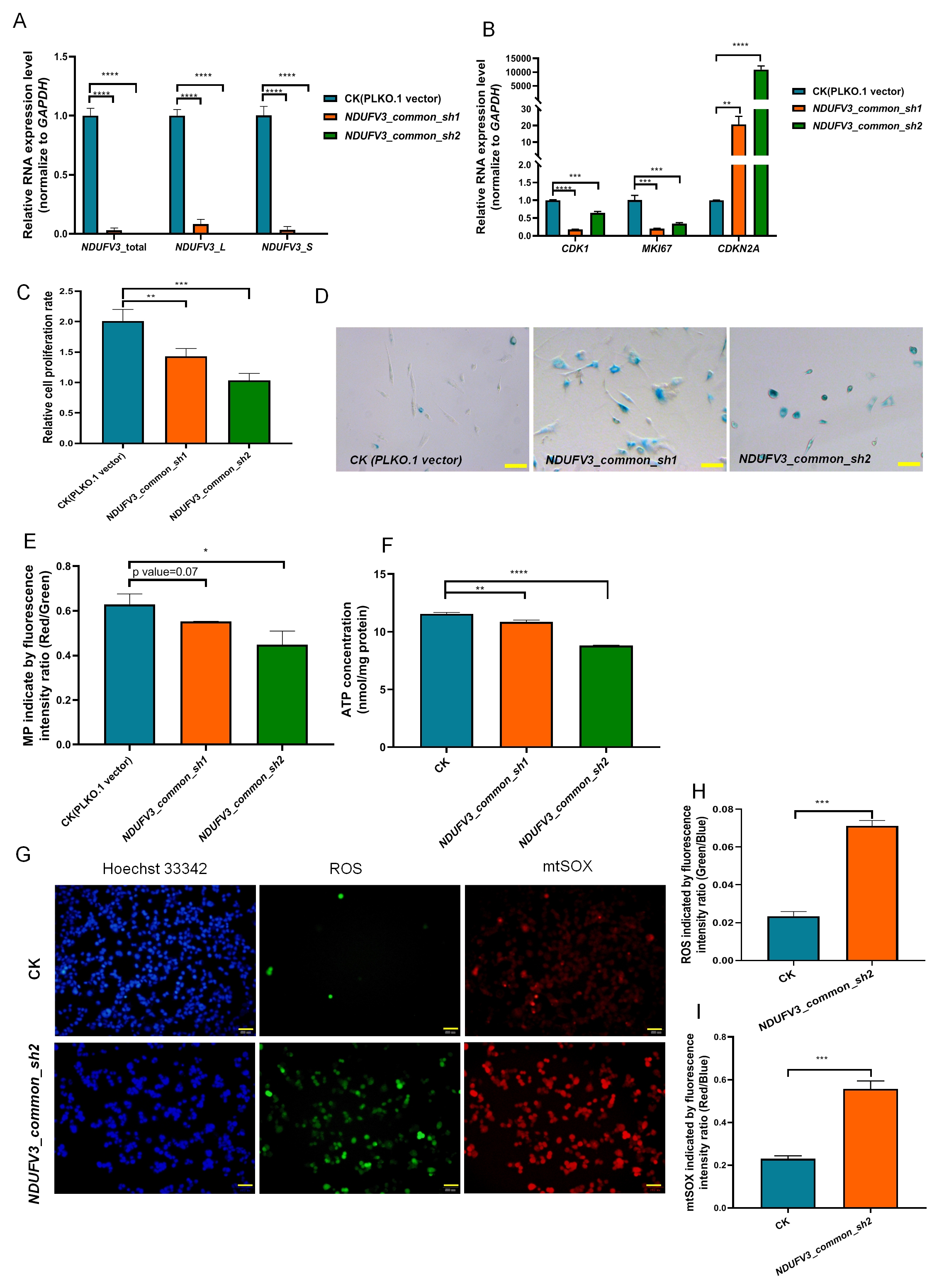


**Figure S7. Knock down of *NDUFV3* can lead to obvious cell senescence phenotypes and impaired mitochondrial functions in HFF and HEK293T cells.**

(A) Gene and transcripts expression levels evaluation of *NDUFV3* before and after *NDUFV3* knock down in HFF cells by qRT-PCR. Note: Due to the high knockdown efficiency, the expression levels of *NDUFV3* and this two transcripts were not detected in in *NDUFV3*_KD HEK293T cells by *NDUFV3_common_sh2*. (B) Gene expression evaluation of *CDK1, MKI67, CDKN2A* before and after *NDUFV3* knock down in HFF cells by qRT-PCR. (C) Relative proliferation rate of control (CK) and *NDUFV3_*KD HFF cells measured by CCK-8 assay. (D) SA-β-Gal staining of control (CK) and *NDUFV3_*KD HFF cells. (E) Quantitative detection of mitochondrial membrane potential by microplate reader based on JC1 staining in control (CK) and *NDUFV3*_KD HFF cells. (F) ATP concentration in control (CK) and *NDUFV3_*KD HEK293T cells. (G) Fluorescence staining images of chromatin (Hoechst 33342), intracellular reactive oxygen species (ROS) and mitochondrial superoxide (mtSOX) in control (CK) and *NDUFV3*_KD HEK293T cells. Bars, 500 μm. (H) Quantitative measure of intracellular reactive oxygen species (ROS) relative amount base on fluorescence intensity ratio (Green/Blue) in control (CK) and *NDUFV3*_KD HEK293T cells. (I) Quantitative measure of intracellular mitochondrial superoxide (mtSOX) relative amount base on fluorescence intensity ratio (Red/Blue) in control (CK) and *NDUFV3*_KD HEK293T cells. *, *p* < 0.05; **, *p* < 0.01; ***, *p* < 0.001; ****, *p* < 0.0001, *t*-test.

## Supplemental Tables

**Table S1. Representative differential exon skipping events in the process of HFF replicative senescence would be explained by the down-regulation of *PTBP1***

| **Gene symbol** | **Position** | **Gene name** |
| --- | --- | --- |
| *ACTN1* | chr14:68878457-68878520 | Actinin Alpha 1 |
| *ACTN1* | chr14:68878988-68879069 | Actinin Alpha 1 |
| *ATP2B4* | chr1:203733222-203733400 | ATPase Plasma Membrane Ca2+ Transporting 4 |
| *BRD8* | chr5:138159554-138159599 | Bromodomain Containing 8 |
| *CCDC25* | chr8:27756718-27756770 | Coiled-Coil Domain Containing 25 |
| *CTSC* | chr11:88328196-88328111 | Cathepsin C |
| *DST* | chr6:56468981-56468999 | Dystonin |
| *EPS15L1* | chr19:16361778-16361984 | Epidermal Growth Factor Receptor Pathway Substrate 15 Like 1 |
| *EPS15L1* | chr19:16377121-16377254 | Epidermal Growth Factor Receptor Pathway Substrate 15 Like 1 |
| *FLOT2* | chr17:28885947-28885856 | Flotillin 2 |
| *GPATCH8* | chr17:44467114-44467096 | G-Patch Domain Containing 8 |
| *HP1BP3* | chr1:20780349-20780540 | Heterochromatin Protein 1 Binding Protein 3 |
| *MACF1* | chr1:39465094-39465112 | Microtubule Actin Crosslinking Factor 1 |
| *METTL26* | chr16:634888-635063 | Methyltransferase Like 26 |
| *NDUFV3* | chr21:42904276-42903181 | NADH:Ubiquinone Oxidoreductase Subunit V3 |
| *PDLIM7* | chr5:177491403-177491420 | PDZ And LIM Domain 7 |
| *PKM* | chr15:72203021-72203188 | Pyruvate Kinase M1/2 |
| *RPS24* | chr10:78040200-78040225 | Ribosomal Protein S24 |
| *SLTM* | chr15:58899468-58899937 | SAFB Like Transcription Modulator |
| *SNHG11* | chr20:38447929-38448093 | Small Nucleolar RNA Host Gene 11 |
| *SPNS1* | chr16:28983785-28983957 | SPNS Lysolipid Transporter 1, Lysophospholipid |
| *TRIP10* | chr19:6746028-6746196 | Thyroid Hormone Receptor Interactor 10 |
| *WAC* | chr10:28595732-28596041 | WW Domain Containing Adaptor with Coiled-Coil |

Note: Such common differential exon skipping events as showing in this table were selected by obvious differences on the figure. This study used the human genome hg38 (GENCODE V40) version provided by UCSC Genome Browser.

**Table S2. Target sequences of shRNAs used in this study**

| **shRNA target gene and ID** | **shRNA target sequence** |
| --- | --- |
| *PTBP1_sh1* | GCGTGAAGATCCTGTTCAATA |
| *PTBP1_sh2* | AACTTCCATCATTCCAGAGAA |
| *PTBP1_sh3* | CTCAACGTCAAGTACAACAAT |
| *NDUFV3_common_sh1* | GACTTGCTTCTACGGTTTCTT |
| *NDUFV3_common_sh2* | GCCGTTTGACAACACTACCTA |
| *NDUFV3_L_sh1* | AGAAAGACTTTGGTAGAGTTT |
| *NDUFV3_L_sh2* | GACAGTTCCTGTTGAGAATAA |
| *NDUFV3_L_sh3* | ACAGCAGCTGAATTGTCTAAA |

**Table S3. qRT-PCR and PCR primers used in this study**

| **Gene and transcript** | **Forward primer sequence** | **Reverse primer sequence** |
| --- | --- | --- |
| *GAPDH* | TTCATTGACCTCAACTACATGGT | CTCAGTGTAGCCCAGGATGCCCTT |
| *PTBP1* | ACCCAGAGAGAGTCACACCC | GTTGCCGTAGTCCTTGGTCA |
| *NDUFV3* | CCAGGTGTTTCGAGGACTTGCT | TGGTGGACTTTGCTTCTTGG |
| *NDUFV3_L* | CCAGGTGTTTCGAGGACTTGCT | CCCTCTCCTTTGGTTCCACTAC |
| *NDUFV3_S* | CCAGGTGTTTCGAGGACTTGCT | CACTGGGGCTGGCTTTTTTGGTGG |
| *CDK1* | AAGCCGGGATCTACCATACC | CCATGTACTGACCAGGAGGG |
| *MKI67* | TCAAGACCCCAGTGAAGGAG | AGATGGCTGTTTTGCTGCAT |
| *LMNB1* | GCGTGCGTGTCTATGCTAAG | CGCCCAGAATCCACCTCTAC |
| *CDKN1A* | GCAGACCAGCATGACAGATTT | AGGAGAACACGGGATGAGGA |
| *CDKN2A* | CTCGTGCTGATGCTACTGAGGA | GGTCGGCGCAGTTGGGCTCC |
| *CDKN2B* | TTTACGGCCAACGGTGGATT | CATCATCATGACCTGGATCGC |
| *NDUFV3_*  *L/S_OE* | gacctccatagaagattctagATGGCTGCCCCGTGTTTGCT | gatccttgcggccgcggatcTCAGTGTCGAGGTGACTCCCG |

Note: Lowercase letters of *NDUFV3_L/S_OE*’s primers represent homologous arm sequences for PCDH_BSD vector. In Fig. 3E, F1 is the common forward primer sequence of *NDUFV3*, *NDUFV3_L* and *NDUFV3_S*; R1 is the reverse primer sequence of *NDUFV3*; R2 is the reverse primer sequence of *NDUFV3_L*; R3 is the reverse primer sequence of *NDUFV3_S*.

**Table S4. Coding sequence of *NDUFV3_L***

| ***NDUFV3_L*** |
| --- |
| ATGGCTGCCCCGTGTTTGCTGCGGCAAGGACGAGCCGGGGCGCTGAAGACTATGCTCCAGGAAGCCCAGGTGTTTCGAGGACTTGCTTCTACGGTTTCTTTGTCTGCGGAATCAGGGAAGAGTGAAAAGGGTCAGCCACAGAATTCCAAGAAGCAAAGTCCACCAAAAAATGTAGTGGAACCAAAGGAGAGGGGCAAGCTCCTAGCCACCCAGACAGCAGCTGAATTGTCTAAAAACTTATCTTCACCCAGTTCTTACCCGCCAGCTGTGAATAAGGGCAGGAAGGTAGCTAGTCCCAGTCCCAGTGGCAGCGTGCTATTCACAGATGAAGGGGTTCCGAAATTTTTGTCAAGAAAGACTTTGGTAGAGTTTCCACAGAAAGTTCTGTCTCCATTCAGAAAACAGGGCTCTGATTCAGAAGCTCGTCAGGTGGGTCGGAAAGTGACGTCGCCTTCGTCTTCATCCTCTTCCAGCTCCTCTGATTCTGAATCTGATGATGAGGCTGACGTTTCAGAGGTCACTCCTCGAGTGGTGAGCAAAGGCAGAGGGGGGCTTCGAAAACCAGAGGCCTCTCATTCCTTTGAAAACAGAGCCCCCCGAGTTACAGTATCAGCAAAAGAGAAAACCTTGCTGCAGAAGCCGCATGTGGACATTACTGATCCAGAGAAGCCCCACCAGCCAAAGAAGAAAGGGTCCCCTGCTAAGCCATCAGAAGGCAGGGAAAATGCGAGACCAAAAACCACAATGCCCAGATCTCAAGTAGATGAAGAGTTTTTGAAGCAAAGTTTAAAGGAAAAACAATTGCAGAAAACATTTAGATTAAATGAAATAGATAAAGAAAGCCAAAAGCCATTTGAAGTTAAAGGACCCTTACCTGTCCACACAAAATCAGGGTTGTCTGCGCCACCGAAGGGCAGCCCAGCGCCTGCTGTGTTGGCAGAAGAGGCCAGAGCAGAGGGGCAGCTGCAAGCCAGTCCTCCTGGGGCGGCAGAGGGGCATCTGGAAAAACCCGTGCCAGAGCCCCAGCGCAAGGCGGCCCCTCCCCTGCCCAGAAAGGAAACCTCAGGGACGCAGGGAATAGAAGGCCACCTGAAGGGTGGACAGGCAATCGTGGAAGATCAGATACCACCAAGCAATTTGGAGACAGTTCCTGTTGAGAATAACCACGGTTTCCATGAAAAGACAGCAGCGCTGAAGCTTGAGGCCGAGGGCGAGGCCATGGAAGATGCAGCCGCGCCAGGGGACGACCGAGGCGGCACACAGGAGCCAGCCCCAGTGCCTGCTGAGCCGTTTGACAACACTACCTACAAGAACCTGCAGCATCATGACTACAGCACGTACACCTTCTTAGACCTCAACCTCGAACTCTCAAAATTCAGGATGCCTCAGCCCTCCTCAGGCCGGGAGTCACCTCGACACTGA |

**Table S5. Coding sequence of *NDUFV3_S***

| ***NDUFV3_S*** |
| --- |
| ATGGCTGCCCCGTGTTTGCTGCGGCAAGGACGAGCCGGGGCGCTGAAGACTATGCTCCAGGAAGCCCAGGTGTTTCGAGGACTTGCTTCTACGGTTTCTTTGTCTGCGGAATCAGGGAAGAGTGAAAAGGGTCAGCCACAGAATTCCAAGAAGCAAAGTCCACCAAAAAAGCCAGCCCCAGTGCCTGCTGAGCCGTTTGACAACACTACCTACAAGAACCTGCAGCATCATGACTACAGCACGTACACCTTCTTAGACCTCAACCTCGAACTCTCAAAATTCAGGATGCCTCAGCCCTCCTCAGGCCGGGAGTCACCTCGACACTGA |
